# Consequences of ‘no-choice, fixed time’ reciprocal host plant switches on nutrition and gut serine protease gene expression in *Pieris brassicae* L. (Lepidoptera: Pieridae)

**DOI:** 10.1371/journal.pone.0245649

**Published:** 2021-01-20

**Authors:** Pawan Kumar, Tabasum Akhter, Parul Bhardwaj, Rakesh Kumar, Usha Bhardwaj, Sudeshna Mazumdar-Leighton

**Affiliations:** Faculty of Science, Department of Botany, University of Delhi, Delhi, India; Laboratoire de Biologie du Développement de Villefranche-sur-Mer, FRANCE

## Abstract

Rapid adaptive responses were evident from reciprocal host-plant switches on performance, digestive physiology and relative gene expression of gut serine proteases in larvae of crucifer pest *P*. *brassicae* transferred from cauliflower (CF, *Brassica oleracea* var. botrytis, family Brassicaceae) to an alternate host, garden nasturtium, (GN, *Tropaeolum majus* L., family Tropaeolaceae) and *vice-versa* under laboratory conditions. Estimation of nutritional indices indicated that larvae of all instars tested consumed the least food and gained less weight on CF-GN diet (significant at p≤0.05) as compared to larvae feeding on CF-CF, GN-GN and GN-CF diets suggesting that the switch to GN was nutritionally less favorable for larval growth. Nevertheless, these larvae, especially fourth instars, were adroit in utilizing and digesting GN as a new host plant type. *In vitro* protease assays conducted to understand associated physiological responses within twelve hours indicated that levels and properties of gut proteases were significantly influenced by type of natal host-plant consumed, change in diet as well as larval age. Activities of gut trypsins and chymotrypsins in larvae feeding on CF-GN and GN-CF diets were distinct, and represented shifts toward profiles observed in larvae feeding continuously on GN-GN and CF-CF diets respectively. Results with diagnostic protease inhibitors like TLCK, STI and SBBI in these assays and gelatinolytic zymograms indicated complex and contrasting trends in gut serine protease activities in different instars from CF-GN diet versus GN-CF diet, likely due to ingestion of plant protease inhibitors present in the new diet. Cloning and sequencing of serine protease gene fragments expressed in gut tissues of fourth instar *P*. *brassicae* revealed diverse transcripts encoding putative trypsins and chymotrypsins belonging to at least ten lineages. Sequences of members of each lineage closely resembled lepidopteran serine protease orthologs including uncharacterized transcripts from *Pieris rapae*. Differential regulation of serine protease genes (Pbr1-Pbr5) was observed in larval guts of *P*. *brassicae* from CF-CF and GN-GN diets while expression of transcripts encoding two putative trypsins (Pbr3 and Pbr5) were significantly different in larvae from CF-GN and GN-CF diets. These results suggested that some gut serine proteases that were differentially expressed in larvae feeding on different species of host plants were also involved in rapid adaptations to dietary switches. A gene encoding nitrile-specifier protein (*nsp)* likely involved in detoxification of toxic products from interactions of ingested host plant glucosinolates with myrosinases was expressed to similar levels in these larvae. Taken together, these snapshots reflected contrasts in physiological and developmental plasticity of *P*. *brassicae* larvae to nutritional challenges from wide dietary switches in the short term and the prominent role of gut serine proteases in rapid dietary adaptations. This study may be useful in designing novel management strategies targeting candidate gut serine proteases of *P*. *brassicae* using RNA interference, gene editing or crops with transgenes encoding protease inhibitors from taxonomically-distant host plants.

## Introduction

The large cabbage white, *P*. *brassicae* is a global pest of crucifer crops. It can also feed on members of plant families Tropaeolaceae, Capparidaceae, Resedaceae, and Papilionaceae [1]. Dietary utilization of diverse plant families by larvae of *Pieris* species implies concurrent mechanisms of behavioral, physiological and developmental adaptations that overcome deleterious effects of characteristic ingested host plant defenses encountered on local and temporal scales [2–6]. Metabolic costs associated with adaptations to complex nutritional intake from a wide range of host plant families can differently affect insect growth, survival, and ultimately, fitness [7,8]. Variable performance of larvae from *Pieris* species on different crucifer and non-crucifer hosts has been reported from natural habitats, agroecosystems and laboratory experiments, worldwide [1,6,9–13]. *P*. *brassicae* larvae are mobile and can move to a new host when the natal host-plant (on which oviposition had occurred) is exhausted. Such mobility is typically coincident with later developmental instars [14,15]. Rapid speed and low metabolic costs in adapting to a dietary change (especially a host plant from a different plant family) implies physiological dexterity of the insect digestive system that needs to be better understood. Studies are available from long-term host plant transfers in Lepidoptera and other insects that demonstrate altered larval performance, accompanied by complex changes in gut transcriptomes when switched to a different species of host plants [6,16,17]. Less is understood of the short-term responses in feeding efficiency and digestive physiology (including molecular changes in gene expression of key players like gut serine proteases) of insects like *P*. *brassicae* larvae in reciprocal switches between host plants that belong to different plant families.

A common mechanism of adaptation to phytophagy and wide diet breadth in several phytophagous pests involves production of insensitive, compensatory and/or degradative proteases in larval guts upon chronic ingestion of host antifeedants, like plant protease inhibitors, PPI [18]. Adaptation to ingested protease inhibitors from different plant families including Brassicaceae and Tropaeolaceae has been reported in larvae of various *Pieris* species [12,19,20]. These plant families also contain distinct classes of glucosinolates that influence herbivory by reacting with plant myrosinases to produce toxic isocyanate moieties, referred to as the “mustard oil bomb” [21,22]. A unique adaptation to herbivory in *Pieris* species involves production of nitrile-specifier protein (NSP) in the larval gut to defuse the “mustard oil bomb” rendering these insects as specialist feeders on plant families containing glucosinolates and in fact, showing preferential oviposition on these hosts [5,23–25]. Mechanisms of adaptation to variable nutrient quality, multiple dietary antifeedants and secondary metabolites in diverse host plant species can influence complex traits like relative larval performance of *Pieris* species at both interspecific and intraspecific levels [12,13,25–28]. While the association of distinct glucosinolates profiles in host plants and larval performance of *Pieris* species is documented [6,10,29] the role of gut serine proteases in rapid dietary adaptations during a reciprocal switch of larvae to an alternate host-plant type is not as well-characterized.

Serine proteases are major insect hydrolases involved in proteolysis of ingested plant proteins as well as adaptation to dietary protease inhibitors [18,30,31]. Biochemical characterization of gut serine protease activities from *Pieris* species feeding on different diets have indicated adaptation to ingested foliar protease inhibitors including Kunitz trypsin inhibitors from *Brassica* species and trypsin/amylase inhibitors from *T*. *majus* [2,12,19,32]. Alteration in suites of gut serine proteases during adaptation to dietary changes has been reported in various phytophagous Lepidoptera within just two hours of exposure to a heterologous PPI [19,33]. It is unclear how long larvae of *P*. *brassicae* have to feed on a new host plant type, before the larval gut proteases fully “sync” with antifeedants encountered on a new diet and optimally utilize ingested food for growth and development. Lepidopteran serine proteases are typically encoded by large gene families and differential expression of genes encoding trypsins, chymotrypsins or other serine proteases of unknown specificity in guts of larvae is a major feature associated with adaptation to polyphagy [13,18,34–36]. The full annotated genome sequence of *P*. *rapae* indicates the presence of a large gene family of putative serine proteases [13]. However, functional characterization of members from the *P*. *brassicae* serine protease gene family that participate in the transition to digestion of a new diet larvae have not been reported. Such proteases may be targeted by novel strategies for controlling insect pests such as RNA interference or CRISPR/Cas9 technologies [37,38] along with transgenic crops expressing heterologous PPI from distantly related host plants [39–41].

In this study. we examined immediate effects of reciprocal host-plant switches between CF and GN diets in actively feeding larvae of *P*. *brassicae* using no-choice, fixed time experiments to assess relative adaptability, nutritional efficiency and larval gut digestive physiology immediately following a dietary change Three developmental stages were examined because larval age can influence adaptation to antifeedants, feeding behavior, and assimilation of dietary nutrients [42–44]. Profiles of larval gut protease activities and their susceptibilities to diagnostic protease inhibitors were determined using a protein, two synthetic substrates and zymography to compare physiological flexibility of *P*. *brassicae* larvae habituated on different host-plant types [12] with larvae from reciprocal host-plant switches. Amplicons obtained using semi-degenerate PCR primers specific for lepidopteran serine protease gene families [33,45] were cloned and sequenced to quickly identify cDNA fragments encoding putative trypsins, chymotrypsins and other serine proteases in gut tissues of fourth instars. Expression of genes encoding these putative gut serine proteases along with a *nsp* gene was examined in fourth instar *P*. *brassicae* from the reciprocal host-plant switch experiments by quantitative real-time PCR to evaluate transitional adaptations to dietary change involving two plant families. Understanding such metabolic switches in the short term can serve as a prelude to understanding long-term insect performance in ecological and evolutionary contexts as well as designing sustainable management strategies for pests like *P*. *brassicae*.

## Materials and methods

### Nutritional indices of larvae switched to alternative hosts in no-choice, fixed time experiments

Host plants, *B*. *oleracea* var. *botrytis* (cauliflower, CF; variety: F1 Hybrid Golden Yellow, India) and *T*. *majus* (garden nasturtium, GN; variety: Jewel Blend, USA) were grown in experimental field plots of the Department of Botany, Delhi University (28.68′ 0′′ North, 77.21′ 0′′ East). Egg clusters were collected from these host plants grown during April-May 2014–17. Neonates hatching from field-collected egg clusters were reared indoors at 25°C ± 3, 70% relative humidity. Provenances for the experimental materials have been previously described [12]. Larval stages were determined from the size of the head capsule measured by a Vernier caliper and freshly molted third, fourth and fifth instars were used for feeding assays. Larvae from the third, fourth and fifth instars for three egg clusters (*n* = 80 ± 9 eggs per egg cluster on CF; 83 ± 6 eggs per egg cluster on GN) were randomly divided into batches, starved for 6–8 hours to empty their gut contents and then shifted to four types of diets: CF-CF, CF-GN, GN-CF and GN-GN, indicating the host on which the insects were feeding before starvation and the host to which the larvae were transferred. The host plants of *B*. *oleracea* corresponding to principal growth stage 4 were used [46] while host plants of *T*. *majus* were at flowering stage. Fresh mature leaves of the two host-plant types were provided to the larvae as described in Kumar et al. [12]. Larvae were fed for 12 hours and parameters *viz*. Plant Weight Consumed (PWC), Larval Weight Gained (LWG), Fecal Matter Produced (FMP) were measured. Approximate Digestibility (AD), Efficiency of conversion of ingested food to body matter (ECI) and Efficiency of conversion of digested food to matter (ECD) [42,47] using following equations: AD = (PWC-FMP)/PWC]*100; ECI = [LWG/PWC]*100; ECD = [LWG]/[PWC-FMP]*100. Appropriate correction factors to account for water loss caused due to detachment of leaf tissues and contribution due to gut-content were also included [12]. Data for percentage values of AD, ECD and ECI were arcsine transformed before performing one-way ANOVA, Tukey’s-HSD (Honestly Significant Difference) using IBM SPSS Statistics ver. 21 (IBM Corporation, NY, USA).

### *In vitro* assays to measure larval gut protease activities and their inhibition by diagnostic inhibitors

Larvae from third, fourth and fifth instars fed on CF-CF, CF-GN, GN-CF and GN-GN diets as described above were used for gut protease assays. For each set of experiments, guts of three larvae were dissected, pooled and homogenized in 500μl of ice cold 0.1M Tris pH 8.0 and centrifuged at 10,000rpm for 5 minutes to obtain clear supernatants for measuring protease activities. Total protease activities were measured using casein substrate at pH 9.0 and trichloroacetic acid (TCA) precipitation-based spectrophotometric assays as described in Bhardwaj et al. [48]. Gut trypsin and chymotrypsin activity were also measured using amidolytic substrates, Nα-benzoyl-D, L-arginine-4-nitroanilide hydrochloride (BApNA, cat #B4875) and N-Succinyl-Ala-Ala-Pro-Phe p-nitroanilide (SAAPFpNA, cat #S7388) respectively as described in Broadway [19]. Susceptibility to plant protease inhibitors was measured using 0.05mM STI (soybean trypsin inhibitor, cat #T9128), 0.125mM SBBI (soybean Bowman-Birk inhibitor, cat #T9777) and 10mM TLCK (tosyl-L-lysine chloromethyl ketone, cat #T7254). The activity of each sample was determined to be the average of three replicates. At least three biological replicates were used for each experiment. Gelatin-zymography was done as per modified protocol of Bhardwaj et al. [48], after Michaud [49] and Oppert et al. [50]. Hydrolytic zones showing proteolytic activity were visible as clear bands against dark blue background. Susceptibility of gut proteases to inhibition by TLCK (5mM) was also examined. All reagents were obtained from Sigma Aldrich Inc., MO, USA. The data from biochemical assays for protease activities were compared by one-way ANOVA as described above.

### RT-PCR, restriction digestions, cloning and sequencing of amplicons encoding putative gut-serine proteases

Gut tissues of fourth instars feeding on CF-CF, CF-GN, GN-CF and GN-GN diets were dissected for isolation of total RNA. The first strand cDNAs were synthesized using anchored Oligo dT_(18)_MN followed by RNA-PCR after Saikia et al. [45]. At least three larvae originating from different egg clusters were used for gut isolations. Primer pairs DmTF/DmTR, DmTF/SerPR and DmTF/RaTR (Table 1) were used for amplification of subsets of putative trypsins, chymotrypsins and other serine protease gene fragments from *P*. *brassicae*. These semi-degenerate primers encode conserved motifs spanning active site residues H_57_ to S_195_ in mammalian serine proteases [51]. The primer SerPR encodes residues C_191_, Q_192_ found in 95% of lepidopteran serine proteases while the primers DmTR and RaTR encode K/R_188_ in addition to D_189_ found in majority of lepidopteran trypsins. Restriction digestions were performed to evaluate the sequence heterogeneity of the RT-PCR products. Restriction digestions of the RT-PCR products were performed using *Alu* I, *Sau* 3AI, *Hae* III and *Hpa* II (New England Biolabs, MA, USA) according to instructions provided by the vendor. RT-PCR products were purified using GeneJET PCR Extraction Kit (Thermo Scientific, cat #K0701, MA, USA) and cloned into pGEM®-T easy vector (Promega Corporation, cat #A1360, WI, USA) as per manufacturers’ instructions. Plasmids containing inserts of expected sizes were sequenced at Macrogen Inc., South Korea using Sanger di-deoxy chain termination method with Big Dye Kit using an ABI3730XL sequencer (Applied Biosystems Inc., CA, USA).

**Table 1:** A list of primers used in this study.

#	Primers	Sequence (5’ to 3)’	Tm (°C)	References
**1**	DmTF	TCGAATTCATTGTGACCGCCGCTCAYTG	63.6	[33,45,52]
2	DmTR	TCTCTAGAGTCACCCTGGCAGGCRTCYTT	59.4	
3	SerPR	TATCTAGATGGGCCACCGGAATCCCCCTG	64.9	
4	RaTR	TCTCTAGAGTCACCCTGGCAGGCRTCYCT	66.5	
5	M13F	GTAAAACGACGGCCAGT	52.6	
6	M13R	AACAGCTATGACCATG	45.4	
7	Pbr1F	ACCGCYGCTCACTGCYTGGCTAAC	65.7	Current study
8	Pbr1R	GCCCTGACATGARGAYTGAGCTGT	61.0	
9	Pbr2F	ACTGGTGACTCTTGGTCGGGAGGAA	63.0	
10	Pbr2R	TCCACAAGTACTGCGGCCGTTACT	62.3	
11	Pbr3F	ACGCTTGGTAAAGAAGCCACTATC	57.1	
12	Pbr3R	GGAATCACCCTTGCAAGAATCTGC	58.5	
13	Pbr4F	GTTGGAACCCAAGCTATCGTATCT	56.6	
14	Pbr4R	CTGRCAGGCRTCCYTTGGTGGATTAAC	62.2	
15	Pbr5F	ACRGTAGCTGGATGGGGTGCGATG	64.0	
16	Pbr5R	GGCRTCYTTATGGGCGGTGGCAGG	66.4	
17	NSP F	GCAAGAAAAGCTTAAGCGCCT	57.6	
18	NSP R	TTCGTCAAATTGGKTYTGRAA	51.8	
19	EF1-α F	ACATTGTCGTCATTGGACAC	53.1	
20	EF1-α R	AGTGTGAAAGCGAGAAGAGC	53.1	
21	ACT F	TCCGATGGTGATNACYTGNCCRTCNGG	64.4	[33]
22	ACT R	CAGGCATCGTGTTGGACTNGGNGAYGGN	67.2	
23	OligodTMA	TTTTTTTTTTTTTTTTTTMA	55.0	[52]
24	OligodTMC	TTTTTTTTTTTTTTTTTTMC		
25	OligodTMG	TTTTTTTTTTTTTTTTTTMG		
26	OligodTMT	TTTTTTTTTTTTTTTTTTMT		

Where Y = C+T; R = A+G; N = A+T+C+G; M = A+C+G.

### Sequence analyses, alignments and tree constructions

After manual trimming of vector regions, consensus sequences of cloned inserts were translated in all six frames to obtain open reading frames (ORFs) for n-BLAST and p-BLAST searches of the non-redundant, NCBI-nr database (www.ncbi.nlm.nih.gov). Sequences of putative serine protease cDNAs expressed in guts of *P*. *brassicae* larvae feeding on cauliflower and *T*. *majus* were submitted to GenBank (S1 Table). Closely related homologous sequences identified from the BLAST results were aligned with CLUSTALW v.2.0.3 in MEGA7.0 [53]. Ontological searches of NCBI databases were used to cull all serine protease sequences of *P*. *rapae* (S1 Table). No sequence homologs of serine proteases with mutations in the canonical active site amino acid residues were used in the analysis. All sequences were trimmed to regions spanning the active site H_57_ and S_195_ residues. No attempt was made to remove paralogs resulting from gene duplication events or alternative splicing isoforms, as the intention was to examine sequence variability of the encoded enzymes. A phylogenetic Bayesian tree was constructed showing relatedness of 208 trimmed serine protease sequences (including one representative of each lineage identified from this study) using BEAST v1.8.3 software [54]. Parameters used were: substitution model: BLOSUM62, site heterogeneity model: Gamma + Invariant sites, Number of gamma categories: 4, tree prior: coalescent: constant and Markov chain Monte Carlo (MCMC): length of change: 10000000. About1000 iterations were used to obtain the best Bayesian tree. Constructed tree was labeled using Fig-Tree (http://tree.bio.ed.ac.uk/software/figtree/).

### Quantitative RT-PCR of cDNAs encoding putative gut serine proteases in larvae switched to alternative hosts

Relative gene expression of five putative gut serine proteases (Pbr1-5) belonging to lineages LI-V was quantified by qRT-PCR. Gene-specific primer pairs (Table 1) were designed from consensus sequences of cloned RT-PCR products representing various lineages of serine proteases expressed in the larval guts (S4 Fig) and validated in end-point RT-PCR before qRT-PCR using equal amounts of total RNAs isolated from fourth instars fed on the different diets. The qRT-PCRs were performed on CFX Connect^TM^ Real-Time PCR Detection System (Bio-Rad Inc., CA, USA) in a total reaction volume of 20μl, containing10μl of iQ^TM^SYBR^®^ Green Supermix (cat #1708882, Bio-Rad Inc., CA, USA), 1μl of the total RNA template (100ng/μl), 1μl of gene-specific forward and reverse primers (150ng/μl) each and 7μl of nuclease-free water at an initial denaturation at 95°C for 1 min, 40 cycles of denaturation at 95°C for 30 seconds, annealing at 55°C for 30 sec and extension at 72°C for 30 sec. A melt curve profile was added at the end of the 40 cycles with a temperature gradient from 65°C to 95°C and an increment of 0.5°C per cycle to ensure homogeneity of each PCR product. Also, primer pairs specific for nitrile specifier protein, NSP (*nsp* gene, GenBank accession KC570465), the elongation factor alpha-1 (*EF1-α* gene; GenBank accession HQ693843) and actin, a housekeeping gene (*act* gene, GenBank accession BBA78421) were used. Transcript levels detected for *act* gene in larval gut samples from CF-CF diets were used to normalize relative fold expression of genes [45,55]. All experiments were performed in triplicates using RNA templates obtained from three independent biological replicates (i.e. larvae from independent egg clusters). The data from qRT-PCR experiments were analyzed by one-way ANOVA and, Tukey’s-HSD (Honestly Significant Difference) at p≤0.05 as described earlier.

## Results

### Host plant type influences nutritional indices of larvae with superior performance of fourth instars on CF-CF diet

**Fig 1:**
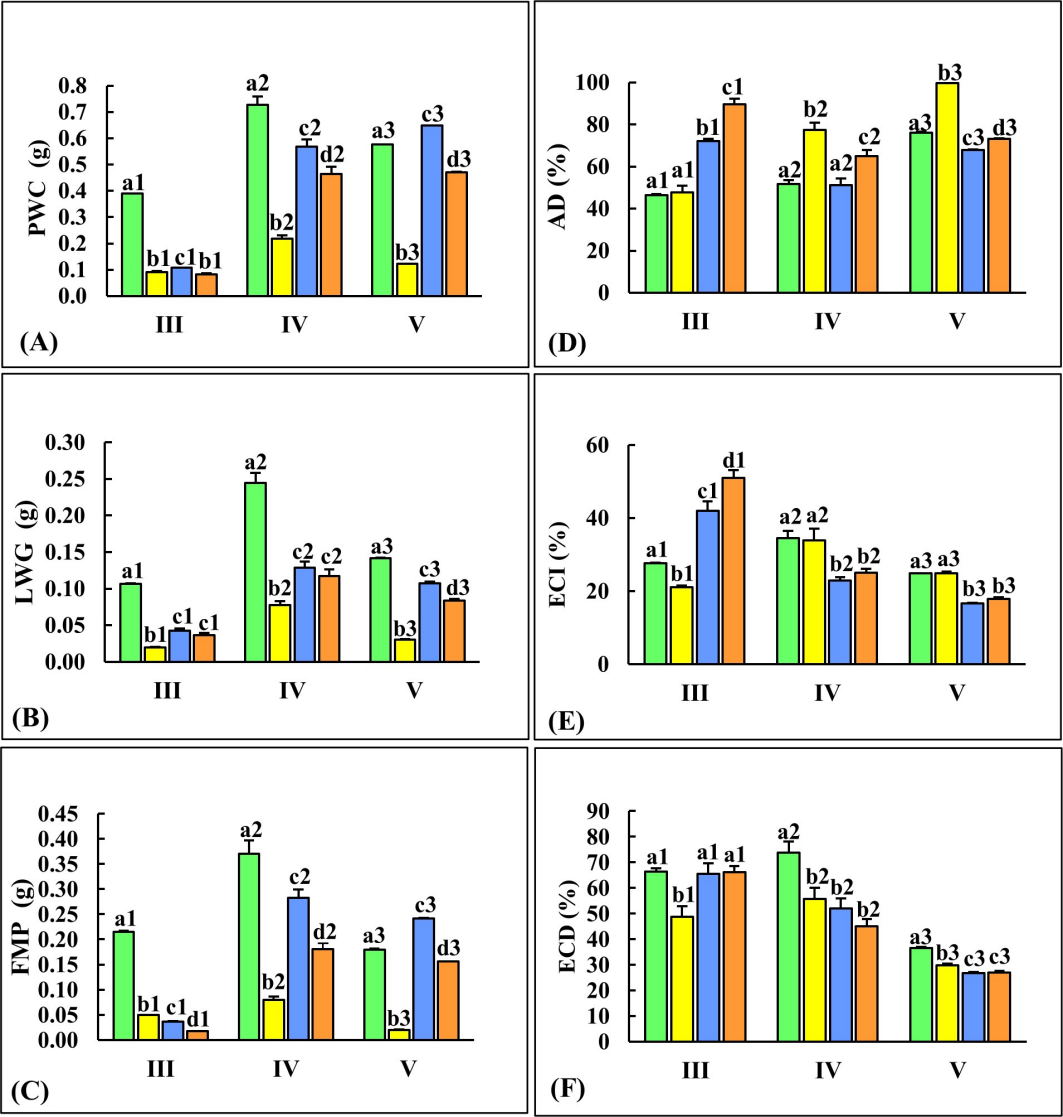
Type of host plant encountered in diet switching experiments influence estimates of nutritional indices in third (III), fourth (IV) and fifth (V) instars from no-choice fixed time, feeding assays. Larvae (n = 27) were reared on CF-CF (green bars), CF-GN (yellow bars), GN-CF (blue bars) and GN-GN (orange bars) diets. Panels shows (A) plant weight consumed (PWC), (B) larval weight gained (LWG), (C) fecal matter produced (FMP), (D) approximate digestibility (AD); (E) efficiency of conversion of ingested food (ECI); and (F) efficiency of conversion of digested food (ECD). Bars shows mean ± SE. Significant differences at p≤0.05 (Tukey’s HSD test) are depicted by different alphabets.

In this study, larvae feeding on the natal host-plants (CF-CF and GN-GN diets) showed significant differences (p≤0.05) in plant weight consumed, larval weight gained and fecal matter produced, with lower values evident in GN-GN fed larvae in all instars (Fig 1A–1C). Larvae from the reciprocal host-plant switch experiments (fed on CF-GN and GN-CF diet) showed major changes in adaptive feeding behavior in the fourth and fifth instars with trends resembling larvae feeding continuously on the host to which it was transferred. Larvae from CF-GN diets showed drastic decrease in plant matter consumed, resembling larvae from GN-GN diets that typically consumed less plant matter than CF-CF fed larvae (Fig 1A). On the other hand, larvae from GN-CF diets consumed more plant matter than GN-GN fed larvae resembling CF-CF diet fed larvae. The CF-GN larvae ate significantly (p≤0.05) less, gained the least amount of weight and produced the least amount of fecal matter indicating poor performance of larvae transferred from cauliflower to *T*. *majus*, in CF-GN diet (Fig 1A–1C). Generally, fourth instars fed CF-CF diets gained the most weight and produced the most fecal matter (Figs 1B and 1C and S1). In comparison to fourth and fifth instars, third instars from all diets (CF-GN, GN-CF, GN-GN diets) ate significantly less (p≤0.05/p≤0.01) plant matter, gained less weight and produced less fecal matter (S1 Fig). Larvae fed GN-GN and GN-CF diets in their fourth and fifth instar showed remarkable increase in the amounts of plant matter consumed (≥ 6 folds), larval weight gained (≥ 2 folds) and fecal matter produced (≥ 6 folds) indicating that older instars were able to successfully utilize GN as a host plant (Figs 1A–1C and S1).

Concomitant effects were observed in measures of nutritional indices from larvae feeding on different diets (Fig 1D–1F). Larvae from the reciprocal host-plant switch experiments (CF-GN and GN-CF diets) showed shifts in estimates of AD and ECD resembling larvae feeding continuously on natal host plants, especially in latter instars. AD in 4^th^ and 5^th^ instars from CF-GN diets were significantly higher than other diets (Fig 1D), suggesting that the small amount of GN matter consumed was digested fully. Because of these high AD values, accompanied by lower ECD values in larvae fed on GN-GN and GN-CF diets, the ECI values were low in these larvae. In case of third instars, ECI; was the highest in larvae fed on GN-GN and GN-CF diets suggesting that metabolic adaptations to host plant type can occur in *P*. *brassicae* during early stages of larval development (Fig 1D). In these ‘no-choice, fixed time’ experiments that explored initial responses of freshly molted larvae to switches in host plant type, estimates of ECI and ECD for insects fed on CF-CF and CF-GN diets from the fourth and fifth stadia were similar to each other, and generally higher than the corresponding larvae fed on GN-GN and GN-CF diets (Fig 1).The highest ECD values were observed for fourth instars fed on CF-CF diets, again indicating that CF was a superior host as compared to GN for *P*. *brassicae* larvae. This result was also supported by highest percentage survival of insects feeding on CF-CF diets in comparison to larvae feeding on other diets from neonate stage to eclosion in field experiments (S2 Fig).

### Altered total gut protease levels in different instars from reciprocal ‘no-choice, fixed time’ host plant switch experiments indicate rapid adaptation to the new diet

**Fig 2:**
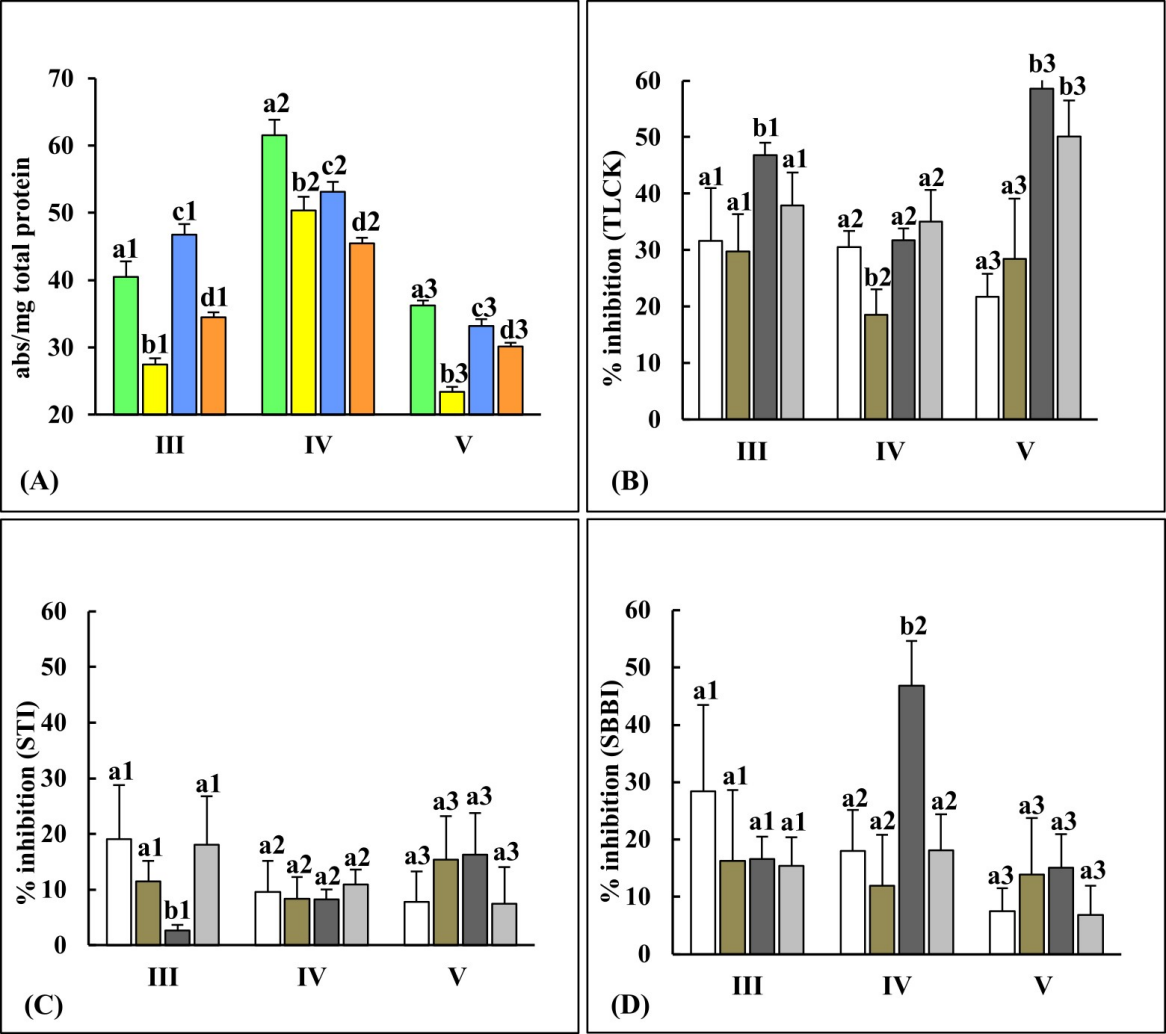
*P*. *brassicae* gut protease activities measured using casein as substrate reveals rapid changes in enzyme levels upon switching to a new diet type. Panel A: Total gut caseinolytic activities detected in III, IV and V instars (n = 36) fed on CF-CF (green), CF-GN (yellow), GN-CF (blue), and GN-GN (orange) diets, in no-choice, fixed time experiments. Inhibition of total protease activity by 3 protease inhibitors (panel B: 10mM TLCK, panel C: 0.05mM STI and panel D: 0.125mM SBBI) is shown. The % inhibition is relative to the protease activity detected in each corresponding gut sample. Bars depict mean ± SE. Significant differences (at p≤0.05, Tukey’s HSD test) are depicted by different letters.

Fig 2A showed that caseinolytic activities detected in gut samples from third, fourth and fifth instars of *P*. *brassicae* were variable when switched to a new diet (CF-GN and GN-CF samples) and when feeding continuously on different species of host plants (CF-CF and GN-GN samples). The levels of total gut protease activities in larvae fed CF-CF diet were significantly different (p≤0.05) from larvae fed GN-GN diet, indicating that proteolytic digestion is influenced by the species of host plant consumed. Fig 2A also showed that levels of gut protease activities in larvae switched to the alternate host plant, (CF-GN and GN-CF diet) were significantly different (p≤0.05) from each other. Gut protease activities were either enhanced or reduced relative to larvae fed control diets (CF-CF or GN-GN). Levels of total protease activities in CF-GN fed larvae was reduced in comparison to CF-CF fed larvae. This reduction was a shift towards the lower levels of proteases detected in GN-GN fed larvae when compared with CF-CF fed larvae. The same scenario was also observed in GN-CF fed larvae whose levels were higher than GN-GN fed larvae, representing a shift towards the high levels of protease activities observed in CF-CF fed larvae. Thus, the general trend was alteration in levels of gut protease in larvae switched between the two host plant types, towards levels of protease activity present in the control diet to which the larvae were switched. These results indicated rapid regulation in levels of larval gut proteases in response to dietary shifts in these host plant switch experiments.

In all three instars tested, gut proteases in larvae feeding on CF (CF-CF and GN-CF diets) were more efficient at proteolysis of casein substrate as compared to gut proteases in GN fed larvae (CF-GN and GN-GN diets). Gut caseinolytic activities were the lowest for larvae fed CF-GN diets in third and fifth instars, indicating that the switch from CF to GN was least adaptive for these insects (Fig 2A). Caseinolytic activities were higher in larvae of all three instars transferred from GN to CF diets as compared to larvae transferred from GN-GN diets (S1 Fig). Interestingly, the highest fold change in protease levels was detected in fourth instars fed CF-GN diets, where enzyme levels were more than twice that observed in the third- and fifth instar (Figs 2A and S1) suggesting differential expression of gut proteases in response to lowered plant food consumption, larval weight gain and fecal matter production (Fig 1A–1C). Total gut protease activities detected in gut samples of larvae fed on each of CF-CF, CF-GN, GN-CF and GN-GN diets were the highest in actively feeding fourth instars and the lowest in fifth instars (S1 Fig).

### Altered gut total trypsin and chymotrypsin levels in different instars from`‘no-choice, fixed time’ reciprocal host-plant switch experiments indicate shift towards proteolytic digestion of new diet

**Fig 3:**
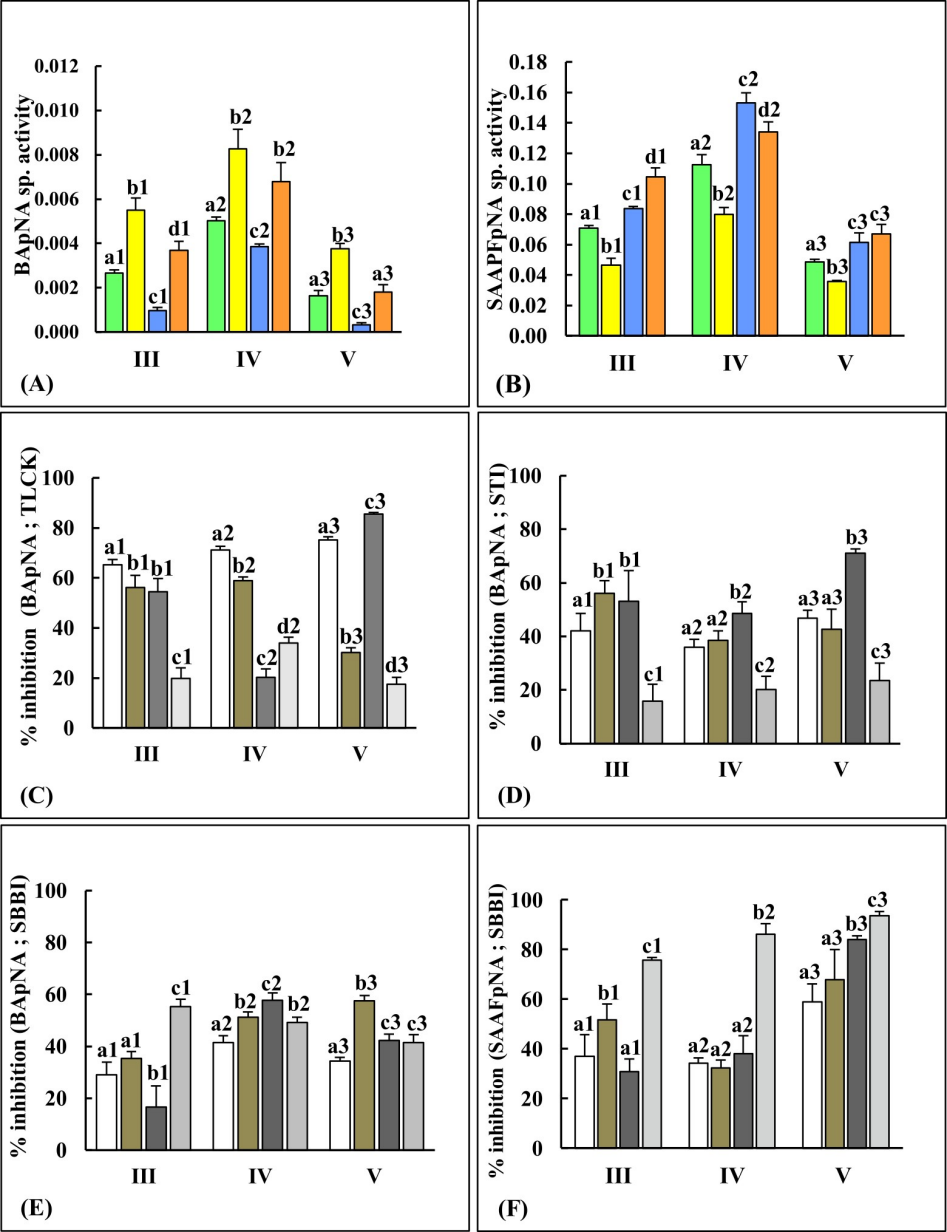
*P*. *brassicae* gut trypsin and chymotrypsin activities measured using amidolytic substrates show different trends in larvae from reciprocal switch experiments. Serine protease activities detected using (A) BApNA and (B) SAAPFpNA substrates in III, IV and V instars (n = 36) fed on CF-CF (green), CF-GN (yellow), GN-CF (blue), and GN-GN (orange) diets in no-choice, fixed time experiments. Inhibition of gut trypsin activity by 3 protease inhibitors are shown (Panel C: 10mM TLCK; Panel D: 0.05mM STI, and Panel E: 0.125mM SBBI) while inhibition of gut chymotrypsin activity by 0.125mM SBBI is shown in panel F. The % inhibition is relative to the trypsin and chymotrypsin activity detected in each corresponding gut sample. Bars depict mean ± SE. Significant differences (at p≤0.05, Tukey’s HSD test) are depicted by different letters.

Levels of gut trypsin activities detected using the amidolytic substrate BApNA were higher in larvae feeding on GN (CF-GN and GN-GN diets) than larvae feeding on CF (Fig 3A). Of the four diets, larvae that were switched to GN (CF-GN diet) had the highest trypsin activities, while larvae switched to CF (GN-CF diet) had significantly lower trypsin activities (p≤0.05//p≤0.01) in each instar (Figs 3A and S1). These results suggested that enhanced levels of trypsin/serine protease activities (detected using BApNA substrate) were likely involved in digestion and/or adaptation to diets containing GN tissues (CF-GN diet). In larvae fed GN-CF diets, reduced levels of gut trypsins were detected which might reflect shift towards the significantly lower levels of trypsins (p≤0.05) in the control CF-CF fed larvae when compared to the control GN-GN fed larvae (Fig 3A).

Among the four sets of diet tested, gut samples ‘originating’ from GN fed larvae (i.e., GN-GN and GN-CF diets) had significantly higher chymotrypsin activities (p≤0.05) than insects originating from CF diets (Fig 3B). However, in contrast to results obtained with gut trypsins, larvae fed on CF-GN diets had significantly reduced levels of gut chymotrypsins in comparison to larvae from the other diets (CF-CF, GN-CF and GN-GN) for all instars tested. In fact, the increase in chymotrypsin activity expected in larvae transferred from CF to GN that would match high levels of chymotrypsin activities observed in larvae from GN-GN diets (expected for digestion of GN tissues) was not evident (Fig 3B). It is not clear if this lack of compensatory response/deficit in chymotrypsin activities in these larvae feeding on CF-GN diet adversely affected the ability of larvae to optimally utilize the GN diet for larval weight gain described above (Fig 1B). In general, trypsin and chymotrypsin activities detected using the two amidolytic substrates were the highest in fourth instars irrespective of the diet (S1 Fig).

### Differential inhibition of gut proteases from different instars in ‘no-choice, fixed time’ reciprocal host-plant switch experiments

Total gut protease activities, trypsin and chymotrypsin activities were inhibited to different extents depending upon the larval age and diet type (Figs 2 and 3). Caseinolytic activities detected in different instars fed on the two host-plant types were inhibited to different extents by TLCK, a small synthetic inhibitor of trypsin (Fig 2B). It inhibited approximately 50% of the gut protease activities in GN-GN and GN-CF fed insects in the third and fifth instars (Fig 2B). Total gut protease activities detected using casein substrate were generally poorly inhibited by STI (<20%) and SBBI (<30%) irrespective of the diet (Fig 2). Gut trypsin and chymotrypsin activities detected in different instars from the four sets of diets were inhibited to varying extents by STI and SBBI (Fig 3). Trypsin activities detected in gut samples of larvae fed GN-CF diets showed the highest degree of inhibition by STI, while trypsins in larvae from GN-GN diets were poorly inhibited by STI in all three instars, highlighting effects of host plant switch on inhibition of gut trypsins (Fig 3D). While TLCK effectively inhibited (>60%) gut trypsins detected in all three instars fed CF-CF diets, gut trypsins in all three instars fed GN-GN diets were poorly inhibited (<40%) by TLCK (Fig 3C). SBBI inhibited approximately 50% of the trypsin-like activities detected in gut samples of larvae fed GN-GN diets (Fig 3E) but inhibited chymotrypsin activities detected in gut samples of larvae fed GN-GN diets from all the three instars by >80% (Fig 3F). In contrast, gut chymotrypsin activities in larvae fed CF-CF diets in the third- and fourth instar were inhibited by only 50% or less by SBBI (Fig 3F). Chymotrypsin activities detected in all gut samples from fifth instar were inhibited by at least 60% and above by SBBI (Fig 3F).

### Qualitative differences in gut-proteases and their inhibition revealed by gelatin-zymography

**Fig 4:**
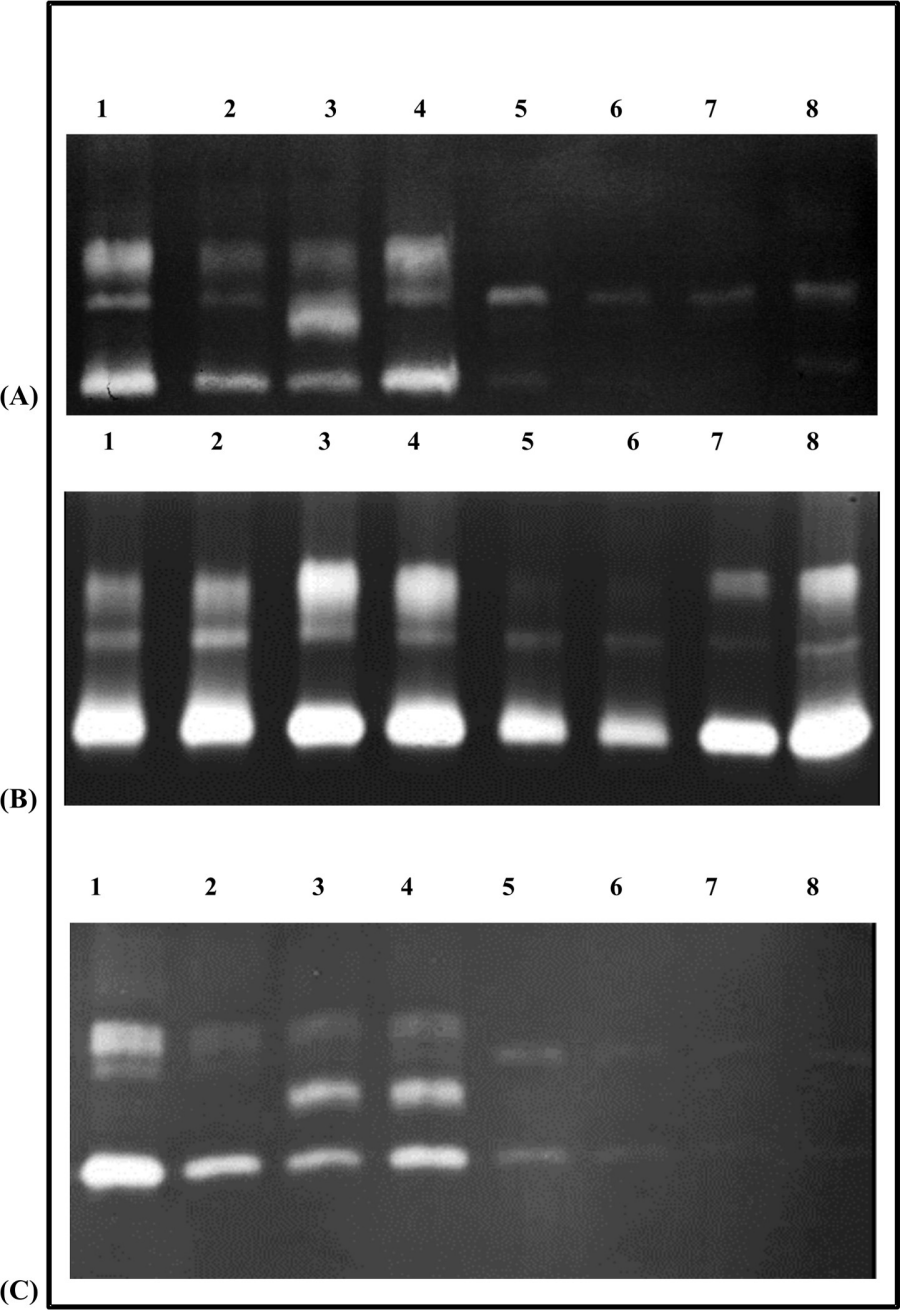
A 12% gelatinolytic zymograms showing different profiles of protease activities in gut extracts of *P*. *brassicae* larvae of different ages fed on different diets. Panels show results for (A) III instars, (B) IV instars, and (C) V instars, feeding on (lane 1) CF-CF, (lane 2) CF-GN, (lane 3) GN-GN, and (lane 4) GN-CF diets, while lanes 5–8 correspond to the same samples incubated with 5mM TLCK.

Activity zones representing proteases of different mobilities were observed in zymograms with gut samples of larvae from third, fourth and fifth instars fed on different diets (Fig 4). The zymograms clearly indicated that incubation with TLCK had a pronounced inhibitory effect on gut proteases of fourth instars from diets of CF origin (CF-CF and CF-GN sets) as opposed to larvae originally feeding on GN (GN-CF and GN-GN sets) (Fig 4B). The presence of slow mobility activity zones containing TLCK-resistant proteases were observed in gut samples of larvae fed GN-CF and GN-GN diets (Fig 4B). Further work is needed to determine if these TLCK-resistant proteases represent gut chymotrypsins. The prominent inhibitory effect of TLCK on trypsins detected in fourth instars fed CF-CF and CF-GN diets (Fig 4B) resembled results from *in vitro* assays (Fig 3C). Fewer activity zones of low intensity in the gelatinolytic zymograms suggested reduced total gut proteolytic activities in third and fifth instars fed on CF-GN diets (Fig 4), corroborating results from the *in vitro* assays described earlier (Fig 2A). Activity zones unique to gut samples of larvae fed GN-GN diet in the third instar (Fig 4A) as well as larvae fed on GN-GN and GN-CF diets in the fifth instar (Fig 4C) were seen, indicating that different types of proteases were produced in different instars depending upon the diet (Fig 4).

### Sequence diversity of gut serine protease cDNAs revealed by RT-PCR, restriction digestions, and analysis of cloned amplicons

In order to study transcripts encoding digestive serine proteases in *P*. *brassicae*, total RNA was isolated from gut tissues of fourth instars feeding on cauliflower (CF) and garden nasturtium *T*. *majus* (GN) diets for 12 hours. Gut cDNAs were synthesized and used for amplification of putative serine protease transcripts. Restriction digestion patterns with various 4 base cutters revealed that the RT-PCR products obtained from gut cDNAs of larvae fed on CF-CF and CF-GN diets were more similar to each other than those from larvae fed GN-GN and GN-CF diets (S3 Fig). Patterns observed from restriction digestions with *Hpa* II and *Alu* I indicated that RT-PCR products obtained from larvae fed on GN-GN and GN-CF diets were also distinct (S3 Fig). BLAST-n and BLAST-p searches confirmed identity of cloned and sequenced RT-PCR amplicons as putative serine proteases (S1 Table). The utility of the semi-degenerate PCR primers for rapid identification of putative trypsins and chymotrypsin gene cDNA fragments expressed in *P*. *brassicae* gut tissues was confirmed from their high sequence homology (>90%) with orthologous genes from *P*. *rapae* (S1 Table). At least ten lineages were inferred from pair-wise sequence identity among the cloned cDNA and designated LI to LX (S4 Fig). The sequence similarity of encoded amino acid sequences varied from 7.4% (between LV and LVII) to 27.1% (between LIV and LVIII), while sequence similarity varied from 52.4% (between LI and LIV) to 66.8% (between LIV and LVII) at the nucleotide level. Members within each lineage showed 91% to 100% sequence identity at the nucleotide level. The closest relatives of *P*. *brassicae* serine proteases from this study were ‘predicted’ serine proteases from *P*. *rapae* (S1 Table).

**Fig 5:**
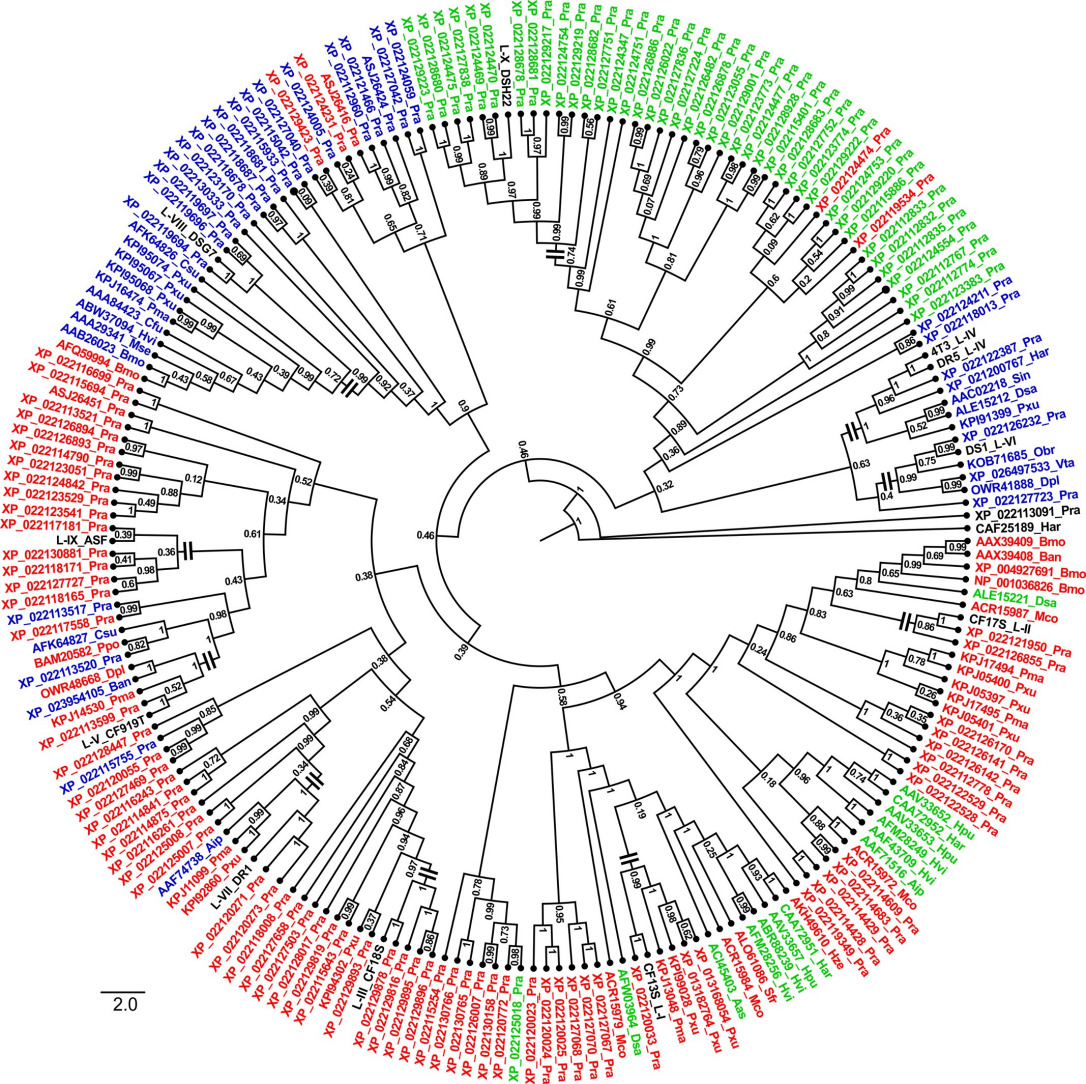
Sequence relatedness of serine proteases identified from *P*. *brassicae*. A Bayesian tree of serine protease sequences spanning from H_57_ until S_195_ (numbering after Bovine chymotrypsinogen, GenBank accession# NP_777139) identified from gut cDNAs of *P*. *brassicae* fed on CF and GN, with related homologs from *P*. *rapae* and various Lepidoptera (S1 Table). Blue markers indicate putative trypsins, green markers indicate putative chymotrypsins while red markers indicate sequences annotated as other serine proteinases. Nodes indicated by a horizontal double bar denote closely-related orthologs from various lineages of gut serine protease genes expressed in *P*. *brassicae* that show ≥50% sequence similarity. The sequence of a putative carboxypeptidase from *H*. *armigera* (GenBank accession# CAF25189) was used as outgroup.

Fig 5 shows a tree depicting sequence relatedness among the gut serine protease cDNAs of *P*. *brassicae*, homologs from *P*. *rapae* as well as several closely related homologs from various Lepidoptera identified from BLAST results. It can be seen that sequences from LI and LII were found on a single large cluster with orthologs from *P*. *rapae* (annotated as serine proteases with collagenase-like functions, S1 Table) and different Lepidoptera (annotated as chymotrypsins). Sequences belonging to LIII and LVII were found in clusters associated with serine proteases like Snake and Easter; while those of LV and LIX are found in clusters with putative trans-membrane serine proteases, TSP and Stubble-like sequences. Sequences of LVIII encoded putative trypsins resembling expressed sequences from various Lepidoptera. A large number of putative chymotrypsin-like sequences from *P*. *rapae* were found in a cluster containing a cDNA from LIX isolated from the hemolymph of *P*. *brassicae*, suggesting immunity-related functions. More work is needed to investigate such a conjecture. The clusters containing sequences from LIV and LVI encoded trypsin and chymotrypsin-like homologs. Interestingly, sequences from LVI cluster included homologs that lack C_191_ (S4 Fig). Multiple sequence alignment of encoded serine proteases from *P*. *brassicae* (S4 Fig) showed the presence of conserved signature motifs including active site residues [56]. While exact specificities of enzymes encoded by these cDNAs needs further examination, lineages III, IV, V, VI, VII, VIII and IX were likely to demonstrate trypsin-like activity due to the occurrence of D_189_ (numbering after bovine chymotrypsin) at the specificity pocket. Serine proteases encoded by two lineages (LI and LII) were likely to show chymotrypsin-like activity. Various other motifs characteristic of serine proteases such as cysteine residues at position 168 and 191; “HP/F/R/S/E/N” at position 91–92 and “GW/F/YG” at position 140–142 respectively were also found in most lineages (S4 Fig). Other than conserved positions typical of serine proteases, the numbers of cysteine residues among the ten lineages were variable.

### *P*. *brassicae* larvae from host-plant switch experiments with cauliflower and garden nasturtium show differential expression of some gut serine protease genes

**Fig 6:**
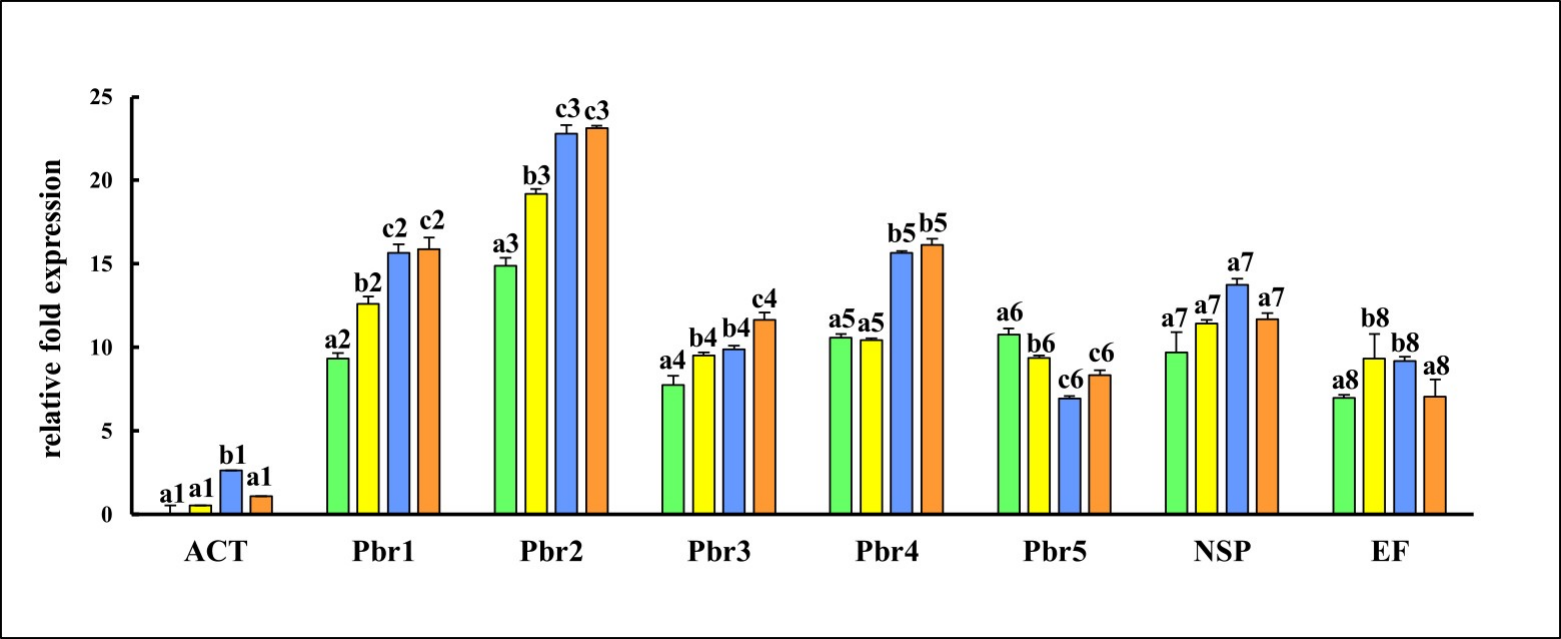
Differential gene expression of some putative serine proteases in *P*. *brassicae* larvae fed on different diets. Relative fold gene expression of gut serine proteases (Pbr1-5) in larvae reared on the following diets: CF-CF (green bars), CF-GN (yellow bars), GN-CF (blue bars) and GN-GN (orange bars). NSP refers to *nsp* gene (accession KC570465) while EF refers to *EF1-α* gene (accession HQ693843). Data were normalized with respect to levels of actin (*act)* gene (accession BBA78421) transcripts detected in larvae fed CF-CF diets. Bars depict mean ± SE. Significant differences (at p≤0.05, Tukey’s HSD test) are denoted by different letters.

Expression levels of Pbr1 (L-I), Pbr2 (L-II), Pbr3 (LIII) and Pbr4 (LIV) genes were significantly higher (p ≤0.05) for insects fed on GN-GN diet relative to those fed on CF-CF diet (Fig 6). Levels of transcripts of Pbr5 were significantly lower in GN-fed insects than CF-fed insects, indicating differential regulation of serine protease genes in response to type of ingested host plant (Fig 6). Transfer from one host type to another (CF-GN and GN-CF samples) resulted in differential gene expression of **Pbr3 and Pbr5** (encoding putative trypsins) as compared to larvae fed on control diets (CF-CF and GN-GN samples). Expression levels of Pbr1, Pbr2, Pbr3 and EF1-α genes were significantly higher (p ≤0.05) in larvae fed on CF-GN diets as compared to larvae fed on control CF-CF diets, indicating rapid changes upon transfer to GN as host (Fig 6). Interestingly, the levels of transcripts of Pbr1, Pbr2, and Pbr4 genes in larvae originating from GN diets (i.e. GN-GN and GN-CF sets) were more similar amongst themselves as compared to larvae originating from CF diets (i.e. CF-CF and CF-GN diets). These genes (encoding two putative chymotrypsins and 1 putative trypsin) were expressed at significantly higher levels in larvae fed on GN-GN and GN-CF diets as compared to larvae from CF-CF and CF-GN diets. These results were reminiscent of high levels of gut chymotrypsins detected in these larvae from GN-GN and GN-CF diets using SAAPFpNA substrate (Fig 3B). The presence of significantly high levels (p ≤0.05) of EF1-α transcripts in larvae fed CF-GN and GN-CF diets suggested active transcription and translation in response to host plant switch. However, the *nsp* gene levels were similar in gut samples from larvae fed CF-CF, CF-GN, GN-CF and GN-GN diets (Fig 6) indicating that variability and/or quantity of ingested glucosinolates found in CF and GN hosts did not influence expression of *nsp* gene. It may be therefore conjectured that while gut serine protease gene expressions were differentially regulated by changes in larval diet, modulation of *nsp* gene levels had only a minor effect on the dietary adaptations to host plant switches observed in the ‘no-choice, fixed time’ experiments in this study.

## Discussion

Pierid butterflies and their interactions with host plants have emerged as model systems to investigate functional adaptations to herbivory [5,6,8,10,13,23,36,57]. Various reports suggest that the ovipositional choice of female *Pieris* butterflies is not always coincident with optimal larval performance on a given host-plant type [15,58,59]. Gregarious, mobile larvae of agricultural pests like *P*. *brassicae* can encounter plants that are similar or different from the natal host plant type. Consequences of such maternal host plant choice (and feeding by early instars) was investigated in mature instars of *P*. *brassicae* fed on cauliflower, (CF-CF diet); garden nasturtium (GN-GN diet) and diets where the natal host-plant type was switched for 12 hours (CF-GN and GN-CF diets). Feeding behavior and nutritional indices like ECD (Fig 1) clearly indicated that CF was a superior host for *P*. *brassicae* larvae as compared to GN. These results were supported by field data (S1 Fig) where percentage survival of neonates until eclosion was the highest on CF host plants (significant at p<0.05) as compared to larvae feeding on GN host plants or switched between the two alternate hosts, CF-GN and GN-CF (S2 Fig). Poor larval performance of *Pieris* species on GN has been associated with nutritional quality and constituent plant chemistry [9,12,60]. Kunitz trypsin inhibitors have been reported from wild and cultivated *Brassicas* as well as *T*. *majus* while foliar thaumatin-like, trypsin/amylase inhibitors such as TpTI have been reported in the latter [12,61,62]. Allyl glucosinolates like sinigrin, are major foliar constituents of crucifer crops like cauliflower and cabbage, while benzyl glucosinolate (glucotropaeolin) is prominent in garden nasturtium [21,57]. Despite differences in nutritional quality of ingested diets *P*. *brassicae* larvae feeding on CF-GN and GN-CF diets were adaptive to dietary changes, exhibiting altered digestibility and physiological plasticity (Fig 1).

Efficient utilization of ingested food plants depends upon the ability of lepidopteran larvae to digest dietary proteins [43,63]. Here, dramatic changes in levels of total protease activities, trypsin and chymotrypsins were observed in the gut of CF-GN and GN-CF diet fed larvae relative to differences observed in larvae fed on natal host plant (CF-CF versus GN-GN samples). Furthermore, gut protease levels in larvae from the switched CF-GN and GN-CF diets showed adaptive trends towards levels observed in larvae feeding on the natal host plants to which they were transferred (Figs 2 and 3). Poor inhibition of gut protease activities in larvae fed on all four diets by STI suggested that these enzymes were already adapted to STI-like Kunitz trypsin inhibitors present in these host plants [2,12]. Gelatinolytic zymograms showing multiple TLCK-resistant protease activity zones (Fig 4), suggested the predominance of chymotrypsins in utilization of GN diets, while susceptibility of gut proteases to TLCK in CF-fed insects indicated predominance of trypsins. These results resembled various reports of the adaptive roles of lepidopteran gut serine proteases, with different participation by trypsins and chymotrypsins in larvae facing complex dietary changes upon chronic ingestion of PPI [12,18,19,34,35,64–66].

This study also indicated a developmental component in physiological adaptations of *P*. *brassicae* larvae to short-term host plant switches, with fourth instars showing most adaptable feeding behaviors and highest gut protease activities on all diets tested (S1 Fig). Similar results have been reported from several Lepidoptera where digestion of ingested plant tissues in the gut improves with larval age and is most versatile in actively feeding penultimate instars preceding pupation [34,67–69]. Results from these reciprocal host plant switch experiments also implied that the length of time spent by larvae feeding on a diet was important to identify proteases that participate in rapid adaptation to dietary shifts. Similar effects of the duration of larval feeding on a given diet for digestive physiology has been reported before [34,65,70]. Generally, for various measures of feeding behavior, AD index, and gut serine protease activities, larvae from reciprocal host plant switch experiments showed a trend towards the natal host plant to which the larvae were transferred. However, for some parameters like ECI, the values showed no change, and resembled the natal host plant from which the larvae were transferred, suggesting that adaptative changes were either delayed or not warranted. For measures in which adaptations towards the new host plant were observed, the extents of upregulation or down-regulation of responses were often strikingly higher than that observed in larvae feeding continuously on the natal hosts. These “excessive” responses may represent a “knee-jerk” reaction to the sudden change in diet, involving a display of physiological plasticity of the insect digestive system.

Occurrence of diverse, multiple genes encoding serine proteases in various Lepidoptera like *P*. *brassicae* may be pivotal to digestive flexibility of larvae during host plant switches where one or more genes can participate in breakdown of ingested plant proteins and/or adaptation to dietary antifeedants like PPI [18,27,35,36,71]. Differential regulation and expression of sequence divergent genes (Fig 5) may reflect the suites of gut serine proteases that participate in physiological adaptations of *P*. *brassicae* larvae to dietary switches. Fig 5 also showed that *P*. *brassicae* and *P*. *rapae* serine protease homologs were found in distinct clusters with high bootstrap support. In fact, these orthologs shared approximately 85.3% (L-II) to 94.8% (L-V) sequence similarity within each lineage at the nucleotide level. The two congeneric species have overlapping dietary preferences [1], suggesting common ancestry for these serine protease genes. If translated, the diverse *P*. *brassicae* serine protease cDNAs identified in this study and their related homologs in *P*. *rapae* from different sequence lineages would likely encode enzymes with different structure and properties (S4 Fig), including susceptibility to PPI. For instance, the nature of amino acids found at positions 90, 148, 151, 188 and 189 in serine proteases is reported to influence susceptibility to ingested plant trypsin inhibitors [35,72,73]. In this study these positions were occupied by variable amino acid residues (S4 Fig). Further work is required to investigate functional diversity among parologs of the pierid serine protease gene family (Fig 5), especially in response to host plant switches and adaptation to dietary constituents like PPI.

Multiple plant defense strategies are dealt with by insects by a variety of adaptive mechanisms, not all of which are significant in influencing larval performance or oviposition [74]. In this study, no significant differences were observed in levels of *nsp* gene transcripts in larvae fed on the four diets for the duration of the experiment (Fig 6). Similar results are available where *nsp* gene levels in *P*. *rapae* are unaltered despite ingestion of tissues differing in levels and/or types of glucosinolates [10,36]. However, further work is required to determine if other members of the *nsp* gene family [25] are differentially expressed during adaptation of *P*. *brassicae* larvae switched between CF and GN in short-term. Global transcriptomic data from larvae of various Lepidoptera does indicate that differential expression of digestive endopeptidases is almost always associated with new host plant use [5,16,27,36]. In this study, differential regulation of only Pbr3 and Pbr5 genes was observed in larvae feeding on CF-GN and GN-CF diets from the reciprocal host-plant switch experiments as opposed to five serine protease genes expressed differentially in the guts of larvae feeding on the natal host plants (Fig 6). While further experiments are required to examine expression levels of genes from other lineages and other instars not tested here, the results indicated that a subset of serine protease genes were more plastic in their response to sudden dietary change involving different host plants (Fig 6). Targeting such specific gut serine protease genes that participate in rapid dietary adaptations may negatively influence larval performance of *P*. *brassicae* on different host plants. These genes would be ideal candidates for knock-out or knock-down experiments [37,38,75,76]. Such efforts would also be enhanced by development of transgenic crucifer crops using genes encoding PPI [77,78] like TpTI from the edible garden nasturtium *T*. *majus*, as a multiplicity of engineered defense mechanisms is likely to provide better management of pests like *P*. *brassicae* that differ in performance on various host plants.

## Supporting information

S1 Fig Nutritional indices and gut protease activities of third (III; green bars), fourth (IV; yellow bars) and fifth (V; blue bars) instars (n = 27 larvae) from fixed time, no choice feeding assays. Larvae were reared on CF-CF, CF-GN, GN-CF and GN-GN diets. Panels show (A) plant weight consumed (PWC), (B) larval weight gained (LWG), (C) fecal matter produced (FMP), (D) gut total caseinolytic activity, (E) gut-trypsin activity and (F) gut-chymotrypsin activity per mg total protein. Bars shows mean ± SE. Significant differences at p≤0.05 (Tukey’s HSD test) are depicted by different alphabets.
(PDF)

S2 Fig Percentage survival of *P*. *brassicae* (n = 6 egg clusters) in reciprocal host plant switch experiments under field conditions. Upon hatching, neonates were immediately transferred from one host plant to the other (CF-GN and GN-CF) and monitored. Percentage survival of neonates until eclosion was measured on caged plants of CF and GN in experimental field slots located at 28.68′ 0′′ N, 77.21′ 0 E. Survivorship was defined as percentage of neonates reaching eclosion. CF-CF (green color); CF-GN (yellow color), GN-CF (blue color) and GN-GN (orange color). Bars depict mean ± SE. Significant differences (at p≤0.05, One-way ANOVA; Tukey’s HSD test) are denoted by different letters.
(PDF)

S3 Fig A 2.5% agarose gel showing *Hpa* II, *Sau* 3AI and *Hae* III digested RT-PCR products amplified from gut tissues of larvae fed on CF-CF (CC), CF-GN (CG), GN-GN (GG) and GN-CF (GC) diets using serine protease-specific primer pairs. (A) DmTF/R and (B) DmTF/SerPR and (C) *Hpa* II, and *Alu* I digested products amplified with DmTF/R from larvae fed on GG and GC diets. Lanes M show 1 kb ladder (Fermentas, USA, catalog# SM0312), and lanes λ show lambda DNA digestion products. Arrows indicate product of ~500bp.
(PDF)

S4 Fig A multiple sequence alignment (MSA) of putative serine proteinases encoded by gut cDNAs of *P*. *brassicae*. Conserved signature motifs are depicted as vertical boxes around the active site residues of H_57_, D_102_ and S_195_ (numbering after bovine chymotrypsin, accession# NP_777139). Sequences of different lineages (roman numerals I-X) are enclosed separately as horizontal boxes. Homologs with the highest sequence similarity to members of each lineage are included in the MSA and shown by their GenBank accession numbers (S1 Table). The symbols “*” refer to fully conserved amino acids along a particular position of a column in the MSA, “:” refers to synonymous changes while “.” refers to similar changes.
(PDF)

S1 Table Sequences of putative serine proteases used for Fig 5. Sequences from this study are shown in bold.
(PDF)

S1 File (PDF)
