# Supplementary material for: Consequences of ‘no-choice, fixed time’ reciprocal host plant switches on nutrition and gut serine protease gene expression in Pieris brassicae L. (Lepidoptera: Pieridae)
Source: PLoS One. 2021 Jan 20;16(1):e0245649. doi: 10.1371/journal.pone.0245649 (PMC7817030; doi:10.1371/journal.pone.0245649)
Supplement: S1 Fig — Larvae were reared on CF-CF, CF-GN, GN-CF and GN-GN diets. Panels show (A) plant weight consumed (PWC), (B) larval weight gained (LWG), (C) fecal matter produced (FMP), (D) gut total caseinolytic activity, (E) gut-trypsin activity and (F) gut-chymotrypsin activity per mg total protein. Bars shows mean ± SE. Significant differences at p≤0.05 (Tukey’s HSD test) are depicted by different alphabets. (PDF) [file pone.0245649.s001.pdf]

S1 Fig

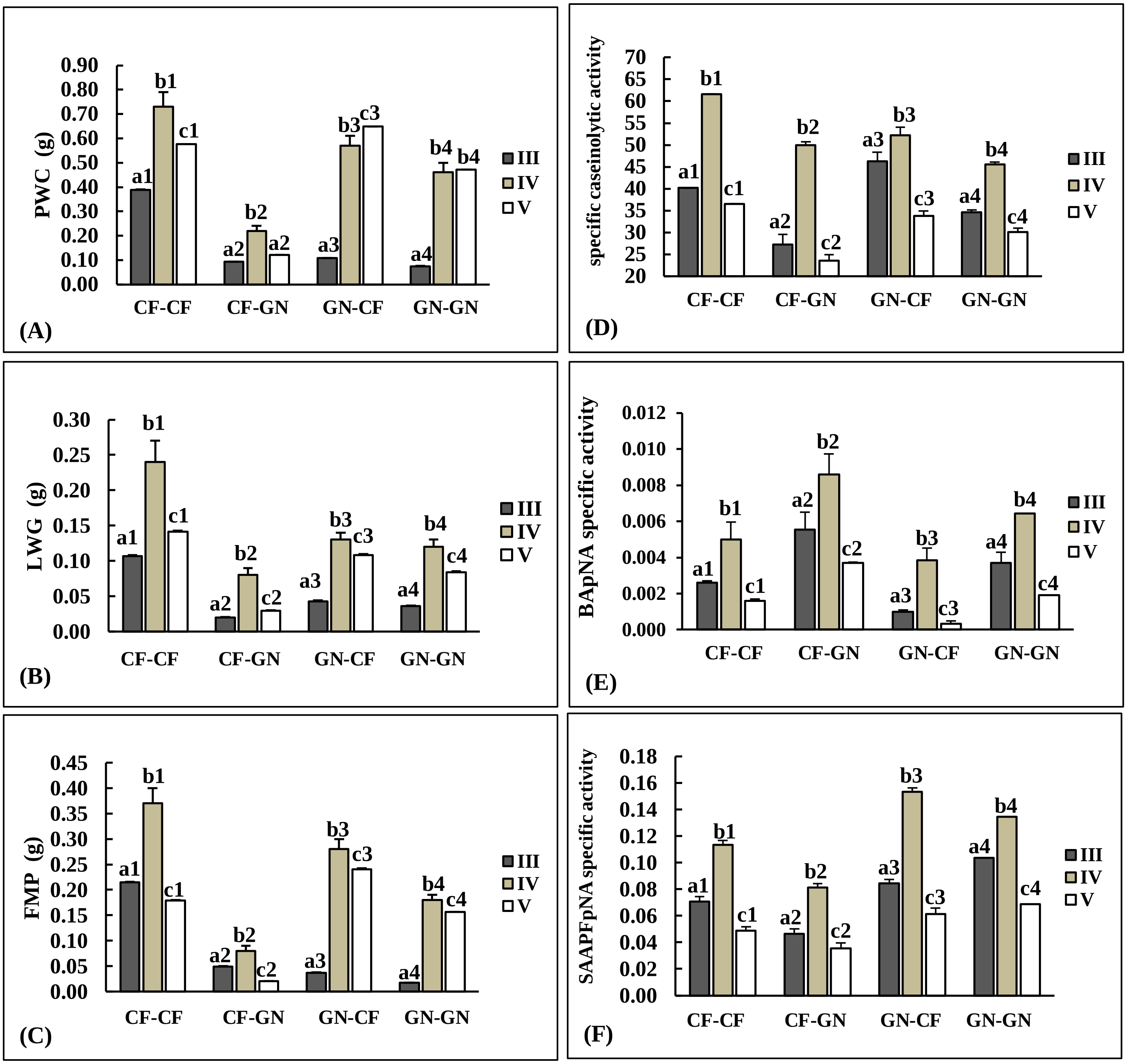

S1 Fig: Nutritional indices and gut protease activities of third (III; black bars), fourth (IV; tan bars) and fifth (V; white bars) instars (n = 27 larvae) from fixed time, no choice feeding assays. Larvae were reared on CF-CF, CF-GN, GN-GN and GN-CF diets. Panels shows (A) plant weight consumed (PWC), (B) larval weight gained (LWG), (C) fecal matter produced (FMP), (D) gut total caseinolytic activity, (E) gut-trypsin activity and (F) gut-chymotrypsin activity per mg total protein. Bars shows mean ± SE. Significant differences at p≤0.05 (Tukey's HSD test) are depicted by different alphabets.
