# Supplementary material for: Consequences of ‘no-choice, fixed time’ reciprocal host plant switches on nutrition and gut serine protease gene expression in Pieris brassicae L. (Lepidoptera: Pieridae)
Source: PLoS One. 2021 Jan 20;16(1):e0245649. doi: 10.1371/journal.pone.0245649 (PMC7817030; doi:10.1371/journal.pone.0245649)
Supplement: S2 Fig — Upon hatching, neonates were immediately transferred from one host plant to the other (CF-GN and GN-CF) and monitored. Percentage survival of neonates until eclosion was measured on caged plants of CF and GN in experimental field slots located at 28.68′ 0′′ N, 77.21′ 0 E. Survivorship was defined as percentage of neonates reaching eclosion. CF-CF (green color); CF-GN (yellow color), GN-CF (blue color) and GN-GN (orange color). Bars depict mean ± SE. Significant differences (at p≤0.05, One-way ANOVA; Tukey’s HSD test) are denoted by different letters. (PDF) [file pone.0245649.s002.pdf]

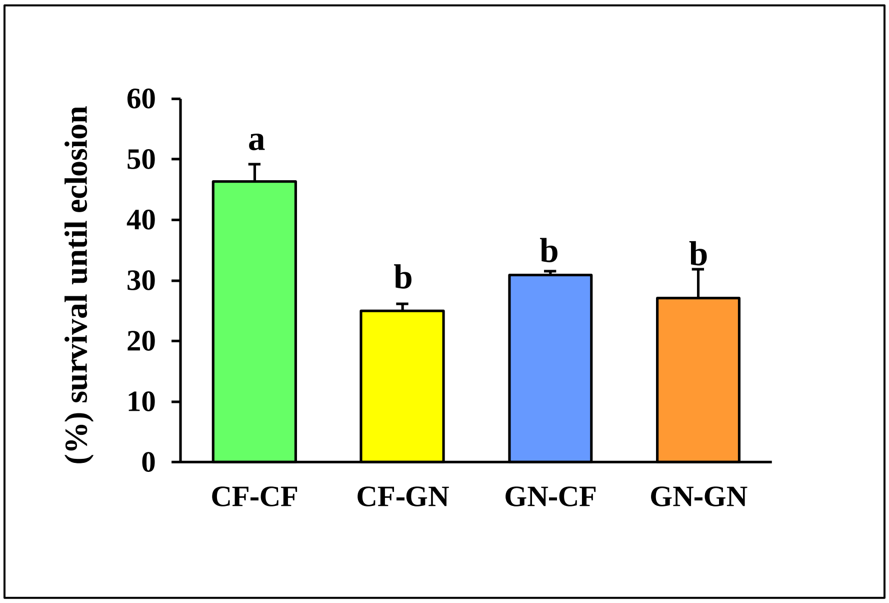

S2 Fig: Percentage survival of *P. brassicae* (n = 6 egg clusters) in reciprocal host-plant switch experiments under field conditions. Upon hatching, neonates were immediately transferred from one host-plant to the other (CF-GN and GN-CF) and monitored. Percentage survival of neonates until eclosion was measured on caged plants of CF and GN in experimental field slots located at 28.68′ 0″ N, 77.21′ 0 E. Survivorship was defined as percentage of neonates reaching eclosion. CF-CF (green color); CF-GN (yellow color), GN-CF (blue color) and GN-GN (orange color). Bars depict mean ± SE. Significant differences (at p≤0.05, One way ANOVA; Tukey's HSD test) are denoted by different letters.
