# Supplementary material for: Consequences of ‘no-choice, fixed time’ reciprocal host plant switches on nutrition and gut serine protease gene expression in Pieris brassicae L. (Lepidoptera: Pieridae)
Source: PLoS One. 2021 Jan 20;16(1):e0245649. doi: 10.1371/journal.pone.0245649 (PMC7817030; doi:10.1371/journal.pone.0245649)
Supplement: S3 Fig — (A) DmTF/R and (B) DmTF/SerPR and (C) Hpa II, and Alu I digested products amplified with DmTF/R from larvae fed on GG and GC diets. Lanes M show 1 kb ladder (Fermentas, USA, catalog# SM0312), and lanes λ show lambda DNA digestion products. Arrows indicate product of ~500bp. (PDF) [file pone.0245649.s003.pdf]

# S3 Fig

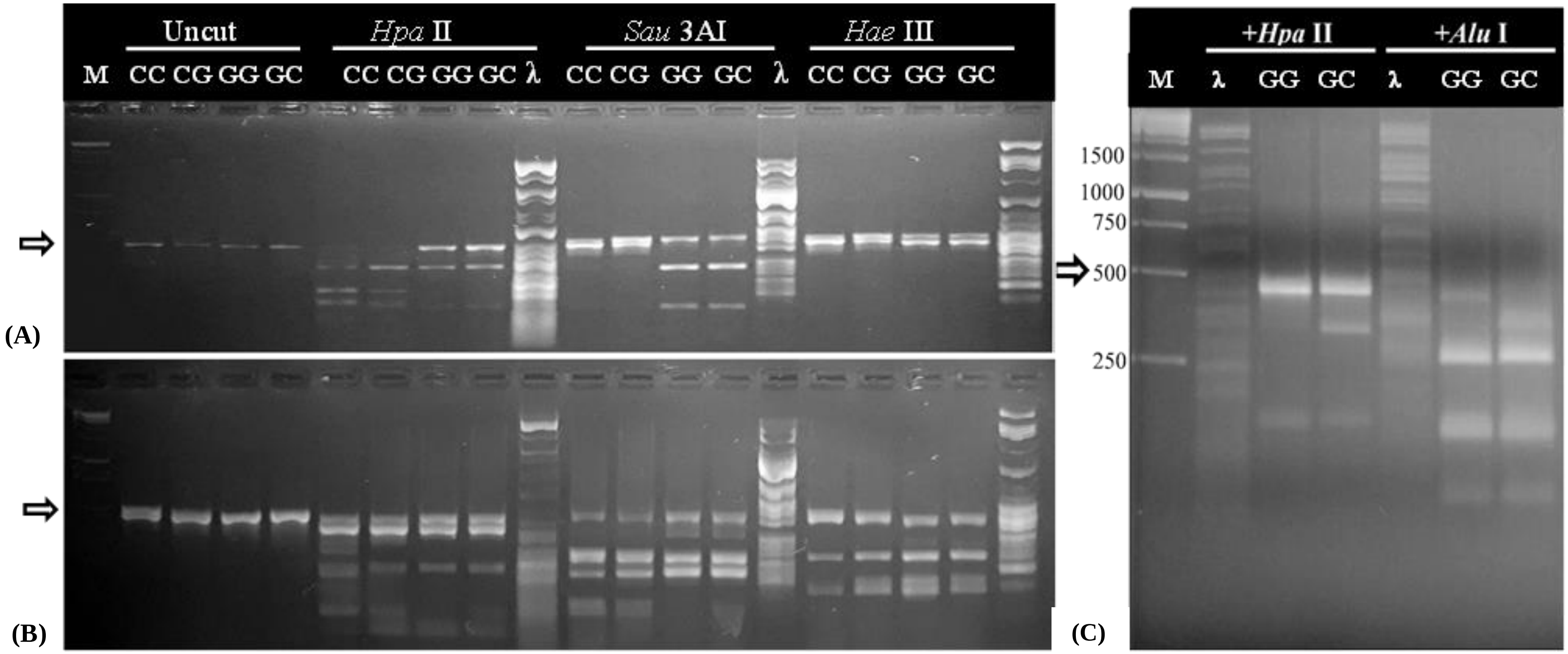


**S3 Fig:** A 2.5% agarose gel showing *Hpa* II, *Sau* 3AI and *Hae* III digested RT-PCR products amplified from gut tissues of larvae fed on CF-CF (CC), CF-GN (CG), GN-GN (GG) and GN-CF (GC) diets using serine protease-specific primer pairs (A) DmTF/R and (B) DmTF/SerPR and (C) *Hpa* II, and *Alu* I digested products amplified with DmTF/R from larvae fed on GG and GC diets. Lanes M show 1 kb ladder (Fermentas, USA, catalog# SM0312), and lanes λ show lambda DNA digestion products. Arrows indicate product of ~500bp.
