# Supplementary material for: Consequences of ‘no-choice, fixed time’ reciprocal host plant switches on nutrition and gut serine protease gene expression in Pieris brassicae L. (Lepidoptera: Pieridae)
Source: PLoS One. 2021 Jan 20;16(1):e0245649. doi: 10.1371/journal.pone.0245649 (PMC7817030; doi:10.1371/journal.pone.0245649)
Supplement: S4 Fig — Conserved signature motifs are depicted as vertical boxes around the active site residues of H57, D102 and S195 (numbering after bovine chymotrypsin, accession# NP_777139). Sequences of different lineages (roman numerals I-X) are enclosed separately as horizontal boxes. Homologs with the highest sequence similarity to members of each lineage are included in the MSA and shown by their GenBank accession numbers (S1 Table). The symbols “*” refer to fully conserved amino acids along a particular position of a column in the MSA, “:” refers to synonymous changes while “.” refers to similar changes. (PDF) [file pone.0245649.s004.pdf]

S4 Fig

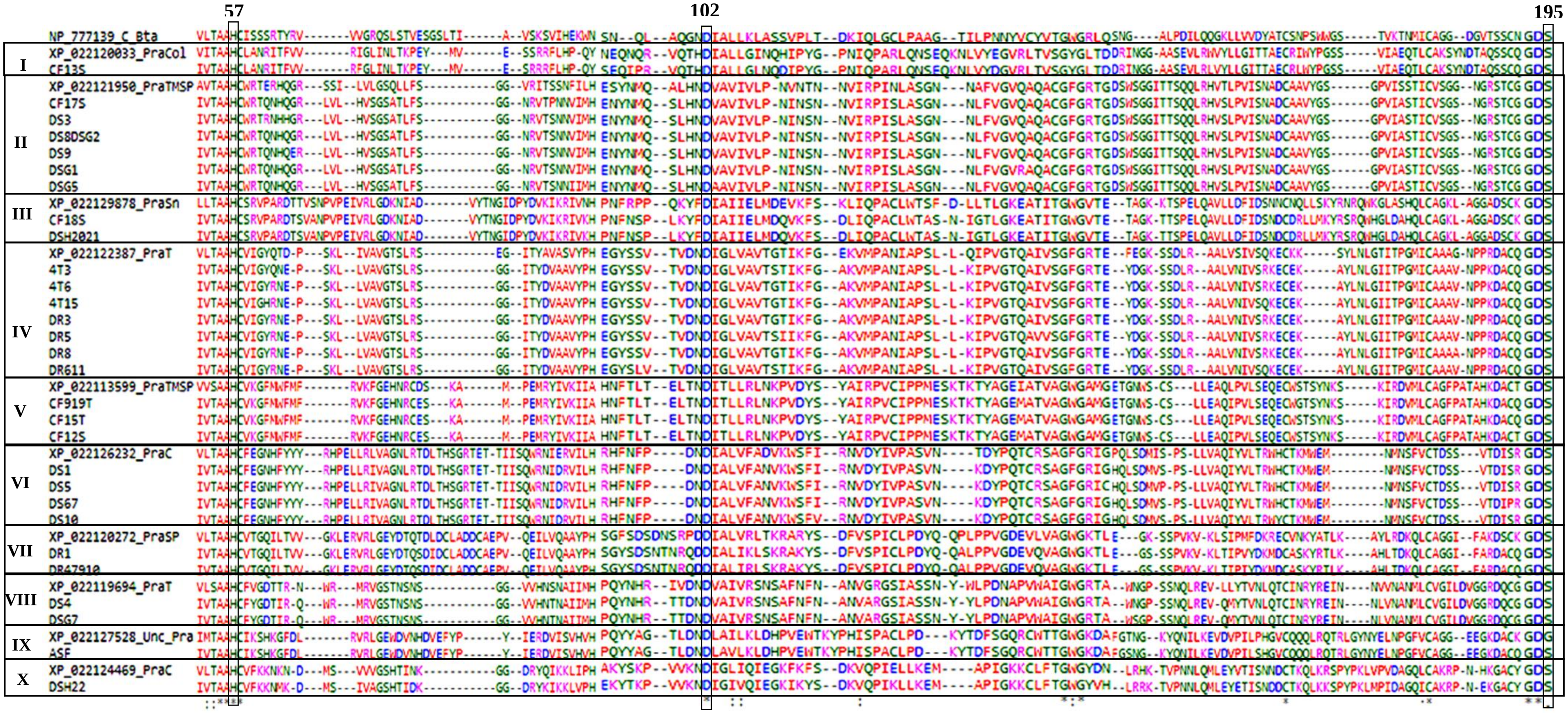

S4 Fig: A multiple sequence alignment (MSA) of putative serine proteinases encoded by gut cDNAs of *P. brassicae*. Conserved signature motifs are depicted as vertical boxes around the active site residues of H_{57}, D_{102} and S_{195} (numbering after bovine chymotrypsin, accession# NP_777139). Sequences of different lineages (roman numerals I-X) are enclosed separately as horizontal boxes. Homologs with the highest sequence similarity to members of each lineage are included in the MSA and shown by their GenBank accession numbers (Table S1). The symbols "*" refer to fully conserved amino acids along a particular position of a column in the MSA, ":" refers to synonymous changes while "." refers to similar changes.
