# Supplementary material for: Consequences of ‘no-choice, fixed time’ reciprocal host plant switches on nutrition and gut serine protease gene expression in Pieris brassicae L. (Lepidoptera: Pieridae)
Source: PLoS One. 2021 Jan 20;16(1):e0245649. doi: 10.1371/journal.pone.0245649 (PMC7817030; doi:10.1371/journal.pone.0245649)
Supplement: S1 Table — Sequences from this study are shown in bold. (PDF) [file pone.0245649.s005.pdf]

S1 Table: Sequences of putative serine proteases used for Fig 5. Sequences from this study are highlighted in bold.

SN	Clone ID/Accesion#	Organism	Herbivore type	Host plant/Family	Descriptions in NCBI Annotations	8 amino-acid signature motif proximal to D189	Tissue source
1	AAV33657	*Helicoverpa punctigera*	Generalist	Nicotiana sp.	Chymotrypsin-like	QSSCQGDS	Gut
2	ABR88239	*Heliothis virescens*	Generalist	*Nicotiana sp.*	Chymotrypsin-like	QSSCQGDS	Midgut
3	ACI45403	*Antheraea assama*	Generalist	*Litsea monopetala*	Putative chymotrypsin	QSSCQGDS	Midgut
4	ACR15979	*Mamestra configurata*	Generalist	*Canola sp.*	Serine protease	QNPCTGDS	Midgut
5	ACR15984	*Mamestra configurata*	Generalist	*Canola sp.*	Serine protease	QSSCQGDS	Midgut
6	AFM28256	*Heliothis virescens*	Generalist	*Nicotiana sp.*	Chymotrypsin	QSSCQGDS	Midgut
7	AFW03964	*Diatraea saccharalis*	Generalist	*Saccharum officinarum*	Chymotrypsin	QSSCQGDS	Midgut
8	AKH49610	*Helicoverpa zea*	Generalist	*Zea mays*	Serine protease 1	QSSCQGDS	Whole organism
9	ALO61086	*Spodoptera frugiperda*	Generalist	*Hordeum sp.*	Serine protease	QASCQGDS	Midgut
10	CAA72951	*Helicoverpa armigera*	Generalist	*Gossypium sp.*	Putative serine protease	QSSCQGDS	Midgut
**11**	**CF13S/QDX19120**	***Pieris brassicae***	**Specialist**	**Crucifers**	**Putative serine protease, partial**	**QSSCQGDS**	**Gut**
12	KPI99028	*Papilio xuthus*	Specialist	Solanaceae	Transmembrane protease serine 9	QSSCGGDS	Whole organism
13	KPJ13048	*Papilio machaon*	Specialist	Solanaceae	Collagenase	QSSCQGDS	Whole organism
14	XP_013168054	*Papilio xuthus*	Specialist	Solanaceae	Predicted: collagenase-like	QSSCQGDS	Whole organism
15	XP_013182764	*Papilio xuthus*	Specialist	Solanaceae	Predicted: collagenase-like	QSSCQGDS	Whole organism
16	XP_022120023	*Pieris rapae*	Specialist	Crucifers	Collagenase-like	QSVCQGDS	Whole organism
17	XP_022120024	*Pieris rapae*	Specialist	Crucifers	Collagenase-like isoform X1	QSVCQGDS	Whole organism
18	XP_022120025	*Pieris rapae*	Specialist	Crucifers	Collagenase-like isoform X2	QSVCQGDS	Whole organism
19	XP_022120033	*Pieris rapae*	Specialist	Crucifers	Collagenase-like	KDTCEGDS	Whole organism
20	XP_022127067	*Pieris rapae*	Specialist	Crucifers	Collagenase-like isoform X1	QSVCQGDS	Whole organism
21	XP_022127068	*Pieris rapae*	Specialist	Crucifers	Collagenase-like isoform X2	QSVCQGDS	Whole organism
22	XP_022127070	*Pieris rapae*	Specialist	Crucifers	Collagenase-like	QSVCQGDS	Whole organism
23	AAF43709	*Heliothis virescens*	Generalist	*Nicotiana* sp.	Chymotrypsin-like	RGTCSGDS	Gut epithelium
24	AAF71516	*Agrotis ipsilon*	Generalist	Multiple hosts	Chymotrypsinogen	RSTCQGDS	Midgut
25	AAV33652	*Helicoverpa punctigera*	Generalist	*Nicotiana* sp.	Chymotrypsinogen	KGTCNGDS	Gut
26	AAV33653	*Helicoverpa*	Generalist	*Nicotiana* sp.	Chymotrypsinogen	KGTCNGDS	Gut

SN	Clone ID/Accesion#	Organism	Herbivore type	Host plant/Family	Descriptions in NCBI Annotations	8 amino-acid signature motif proximal to D189	Tissue source
		punctigera					
27	AAX39408	*Bombyx mandarina*	Specialist	*Morus alba*	Serine protease	RSTCSGDS	Midgut
28	AAX39409	*Bombyx mori*	Specialist	*Morus alba*	Serine protease	RSTCRGDS	Midgut
29	ACR15972	*Mamestra configurata*	Generalist	*Canola sp.*	Serine protease	RSTCNGDS	Midgut
30	ACR15987	*Mamestra configurata*	Generalist	*Canola sp.*	Serine protease	VGTCGGDS	Midgut
31	AFM28249	*Heliothis virescens*	Generalist	*Nicotiana sp.*	Chymotrypsin	RGTCSGDS	Midgut
32	ALE15221	*Diatraea saccharalis*	Generalist	*Saccharum officinarum*	Chymotrypsin	NSPCPGDS	Midgut
33	CAA72952	*Helicoverpa armigera*	Generalist	*Gossypium sp.*	Putative serine protease	KGTCNGDS	Midgut
**34**	**CF17S/QDX19121**	***Pieris brassicae***	**Specialist**	**Crucifers**	**Putative serine protease, partial**	**RSTCGGDS**	**Gut**
35	KPJ05397	*Papilio xuthus*	Specialist	Solanaceae	Collagenase	VSTCGGDS	Whole organism
36	KPJ05400	*Papilio xuthus*	Specialist	Solanaceae	Collagenase	RSTCGGDS	Whole organism
37	KPJ05401	*Papilio xuthus*	Specialist	Solanaceae	Collagenase	ISTCSGDS	Whole organism
38	KPJ17494	*Papilio machaon*	Specialist	Solanaceae	Collagenase	RSTCGGDS	Whole organism
39	KPJ17495	*Papilio machaon*	Specialist	Solanaceae	Collagenase	ISTCHGDS	Whole organism
40	NP_001036826	*Bombyx mori*	Specialist	*Morus alba*	Serine protease	RSTCSGDS	Wing disc
41	XP_004927691	*Bombyx mori*	Specialist	*Morus alba*	Collagenase	RSVCSGDS	Whole organism
42	XP_022112778	*Pieris rapae*	Specialist	Crucifers	Collagenase-like	ASTCAGDS	Whole organism
43	XP_022114428	*Pieris rapae*	Specialist	Crucifers	Collagenase-like	VGTCEGDS	Whole organism
44	XP_022114429	*Pieris rapae*	Specialist	Crucifers	Collagenase-like	TSTCQGDS	Whole organism
45	XP_022114609	*Pieris rapae*	Specialist	Crucifers	Transmembrane protease serine 9-like	KSTCRGDS	Whole organism
46	XP_022114683	*Pieris rapae*	Specialist	Crucifers	Brachyurin-like	KDACQGDS	Whole organism
47	XP_022119349	*Pieris rapae*	Specialist	Crucifers	Collagenase-like	SSACSGDS	Whole organism
48	XP_022121950	*Pieris rapae*	Specialist	Crucifers	Transmembrane protease serine 9-like	RDACQGDS	Whole organism
49	XP_022122528	*Pieris rapae*	Specialist	Crucifers	Collagenase-like	IGTCQGDS	Whole organism
50	XP_022122529	*Pieris rapae*	Specialist	Crucifers	Collagenase-like	VDACQGDS	Whole organism
51	XP_022126141	*Pieris rapae*	Specialist	Crucifers	Collagenase-like	ASTCGGDS	Whole organism
52	XP_022126142	*Pieris rapae*	Specialist	Crucifers	Collagenase-like	RSTCGGDS	Whole organism
53	XP_022126170	*Pieris rapae*	Specialist	Crucifers	Collagenase-like	TDISRGDS	Whole organism
54	XP_022126855	*Pieris rapae*	Specialist	Crucifers	Collagenase-like	RSTCGGDS	Whole organism

SN	Clone ID/Accesion#	Organism	Herbivore type	Host plant/Family	Descriptions in NCBI Annotations	8 amino-acid signature motif proximal to D189	Tissue source
**55**	**CF18S/QDX19122**	***Pieris brassicae***	**Specialist**	**Crucifers**	**Putative serine protease, partial**	**ADSCKGDS**	**Gut**
56	KPI94302	*Papilio xuthus*	Specialist	Solanaceae	Serine protease snake	IDACQGDS	Whole organism
57	XP_022115643	*Pieris rapae*	Specialist	Crucifers	Serine protease Hayan-like	KDACQGDS	Whole organism
58	XP_022127503	*Pieris rapae*	Specialist	Crucifers	Uncharacterized LOC111001791	KDTCQGDS	Whole organism
59	XP_022127658	*Pieris rapae*	Specialist	Crucifers	Serine protease persephone-like	HDTCQGDS	Whole organism
60	XP_022128017	*Pieris rapae*	Specialist	Crucifers	Serine protease snake-like isoform X1	KDTCQGDS	Whole organism
61	XP_022129816	*Pieris rapae*	Specialist	Crucifers	Serine protease snake-like	VDACQGDS	Whole organism
62	XP_022129819	*Pieris rapae*	Specialist	Crucifers	Serine protease snake-like	VDTCQGDS	Whole organism
63	XP_022129878	*Pieris rapae*	Specialist	Crucifers	Serine protease snake-like	ADSCKGDS	Whole organism
64	XP_022129893	*Pieris rapae*	Specialist	Crucifers	Serine protease snake-like	VDTCQGDS	Whole organism
65	XP_022129895	*Pieris rapae*	Specialist	Crucifers	Serine protease snake-like	VDACQGDS	Whole organism
66	XP_022129896	*Pieris rapae*	Specialist	Crucifers	Serine protease snake-like	VDACQGDS	Whole organism
**67**	**4T3/AYN79794**	***Pieris brassicae***	**Specialist**	**Crucifers**	**Trypsin, partial**	**KDACQGDS**	**Gut**
68	AAC02218	*Scirpophaga incertulas*	Specialist	*Oryza* sp.	Putative trypsin	KDACQGDS	Midgut
69	ALE15212	*Diatraea saccharalis*	Generalist	*Saccharum officinarum*	Trypsin-like	RDACQGDS	Midgut
70	KPI91399	*Papilio xuthus*	Specialist	Solanaceae	Trypsin-1	RDACQGDS	Whole organism
**71**	**DR6/QDX19119**	***Pieris brassicae***	**Specialist**	**Crucifers**	**Putative serine protease, partial**	**RDACQGDS**	**Gut**
72	XP_021200767	*Helicoverpa armigera*	Generalist	*Gossypium sp.*	Trypsin-3-like	RDACQGDS	Whole organism
73	XP_022122387	*Pieris rapae*	Specialist	Crucifers	Trypsin-1-like	VDACRGDS	Whole organism
**74**	**CF919T/QDX19124**	***Pieris brassicae***	**Specialist**	**Crucifers**	**Putative trypsin, partial**	**KDACQGDS**	**Gut**
**75**	**DS1/QDX19117**	***Pieris brassicae***	**Specialist**	**Crucifers**	**Putative serine protease, partial**	**TDISRGDS**	**Gut**
76	KOB71685	*Operophtera brumata*	Generalist	Multiple Hosts	Transmembrane serine protease 9	SDVAMGDS	Head and Thorax
77	OWR41888	*Danaus plexippus plexippus*	Specialist	Apocynaceae	Chymotrypsinogen A	SDVSRGDS	Whole organism
78	XP_022126232	*Pieris rapae*	Specialist	Crucifers	Chymotrypsinogen A-like	TDISRGDS	Whole organism
79	XP_026497533	*Vanessa tameamea*	Generalist	Multiple Hosts	Chymotrypsin-1-like	TDVSRGDS	Thorax
80	AAF74738	*Agrotis ipsilon*	Generalist	Poaceae	Trypsin precursor	KDACQGDS	Midgut
**81**	**DR1/QDX19117**	***Pieris brassicae***	**Specialist**	**Crucifers**	**Putative trypsin, partial**	**RDACQGDS**	**Gut**
82	KPI92860	*Papilio xuthus*	Specialist	Solanaceae	Serine protease Easter	EDACRGDS	Whole organism
83	KPJ11099	*Papilio machaon*	Specialist	Solanaceae	Serine protease Easter	EDACRGDS	Whole organism

SN	Clone ID/Accesion#	Organism	Herbivore type	Host plant/Family	Descriptions in NCBI Annotations	8 amino-acid signature motif proximal to D189	Tissue source
84	XP_022114841	*Pieris rapae*	Specialist	Crucifers	Melanization protease 1-like partial	KDSCNGDS	Whole organism
85	XP_022114875	*Pieris rapae*	Specialist	Crucifers	Serine protease Easter-like	IDGCFGDS	Whole organism
86	XP_022116243	*Pieris rapae*	Specialist	Crucifers	Serine protease Easter-like	QDSCRGDS	Whole organism
87	XP_022116261	*Pieris rapae*	Specialist	Crucifers	Hepatocyte growth factor activator-like	KDTCQGDS	Whole organism
88	XP_022120271	*Pieris rapae*	Specialist	Crucifers	Serine protease 7-like isoform X1	KDSCKGDS	Whole organism
89	XP_022120273	*Pieris rapae*	Specialist	Crucifers	Serine protease 7-like	TSVCNGDS	Whole organism
90	XP_022125007	*Pieris rapae*	Specialist	Crucifers	Serine protease Easter-like	KDSCKGDS	Whole organism
91	XP_022125008	*Pieris rapae*	Specialist	Crucifers	Serine protease Easter-like	ISACNGDS	Whole organism
92	AAA29341	*Manduca sexta*	Specialist	Solanaceae	Alkaline midgut trypsin	RDQCQGDS	Midgut
93	AAA84423	*Choristoneura fumiferana*	Specialist	Pinaceae	Trypsin gene	RDQCQGDS	Gdna
94	AAB26023	*Bombyx mori*	Specialist	*Morus alba*	Trypsin-like protease	RDQCQGDS	Midgut
95	ABW37094	*Heliothis virescens*	Generalist	*Nicotiana sp.*	Putative trypsin-like	RDQCQGDS	Midgut
96	AFK64826	*Chilo suppressalis*	Specialist	*Oryza sativa*	Trypsin-like proteinase	RDQCTMDS	Midgut & hemolymph
97	AFQ59994	*Bombyx mori*	Specialist	*Morus alba*	Serine protease	RDQCQGDS	Midgut
98	ASJ26416	*Pieris rapae*	Specialist	*Crucifers*	Seminal fluid protein	KDSCQGDS	Male ejaculate
99	ASJ26424	*Pieris rapae*	Specialist	*Crucifers*	Trypsin-like serine protease partial	KGACFGDS	Male ejaculate
100	KPI95067	*Papilio xuthus*	Specialist	Solanaceae	Trypsin CFT-1	RDQCQGDS	Whole organism
101	KPI95068	*Papilio xuthus*	Specialist	Solanaceae	Trypsin CFT-1-like	RDQCQGDS	Whole organism
102	KPI95074	*Papilio xuthus*	Specialist	Solanaceae	Trypsin CFT-1	RDQCQGDS	Whole organism
103	KPJ16474	*Papilio machaon*	Specialist	Solanaceae	Trypsin CFT-1	RDQCQGDS	Whole organism
**104**	**DSG7/QDX19128**	***Pieris brassicae***	**Specialist**	**Crucifers**	**Putative serine protease, partial cds**	**RDQCGGDS**	**Gut**
105	XP_022112960	*Pieris rapae*	Specialist	Crucifers	Trypsin-like	KDSCQGDS	Whole organism
106	XP_022115042	*Pieris rapae*	Specialist	Crucifers	Trypsin alkaline C-like	SGPCLGDS	Whole organism
107	XP_022115933	*Pieris rapae*	Specialist	Crucifers	Trypsin-like isoform X2	KDSCGGDS	Whole organism
108	XP_022118678	*Pieris rapae*	Specialist	Crucifers	Trypsin alkaline C-like	RDACQGDS	Whole organism
109	XP_022118681	*Pieris rapae*	Specialist	Crucifers	Trypsin alkaline B-like	RDACQRDS	Whole organism
110	XP_022118687	*Pieris rapae*	Specialist	Crucifers	Trypsin alkaline B-like	DNVCSSDS	Whole organism
111	XP_022119694	*Pieris rapae*	Specialist	Crucifers	Trypsin	RDQCGGDS	Whole organism
112	XP_022119696	*Pieris rapae*	Specialist	Crucifers	Trypsin CFT-1-like	RNQCVGDS	Whole organism

SN	Clone ID/Accesion#	Organism	Herbivore type	Host plant/Family	Descriptions in NCBI Annotations	8 amino-acid signature motif proximal to D189	Tissue source
113	XP_022119697	*Pieris rapae*	Specialist	Crucifers	Trypsin CFT-1-like	QSVCQGDS	Whole organism
114	XP_022121466	*Pieris rapae*	Specialist	Crucifers	Trypsin 5G1-like	RSTCGGDS	Whole organism
115	XP_022123170	*Pieris rapae*	Specialist	Crucifers	Trypsin alkaline B-like	EGTCHGDS	Whole organism
116	XP_022124005	*Pieris rapae*	Specialist	Crucifers	Trypsin-2-like	ESTCQGDS	Whole organism
117	XP_022124059	*Pieris rapae*	Specialist	Crucifers	Trypsin-3-like	IAACSGDS	Whole organism
118	XP_022124231	*Pieris rapae*	Specialist	Crucifers	Transmembrane protease serine 9-like	RVACNGDS	Whole organism
119	XP_022127040	*Pieris rapae*	Specialist	Crucifers	Trypsin-7-like	PDACQGDS	Whole organism
120	XP_022127042	*Pieris rapae*	Specialist	Crucifers	Trypsin 3A1 isoform X2	VSSCQGDS	Whole organism
121	XP_022129423	*Pieris rapae*	Specialist	Crucifers	Vitellin-degrading protease-like	KDACQGDS	Whole organism
122	XP_022130333	*Pieris rapae*	Specialist	Crucifers	Trypsin CFT-1-like	KNTCFGDS	Whole organism
123	AFK64827	*Chilo suppressalis*	Specialist	*Oryza sativa*	Trypsin-like proteinase	KDSCQGDS	Midgut
124	ASJ26451	*Pieris rapae*	Specialist	*Crucifers*	Serine protease like partial	KDACQGDS	Male ejaculate
125	BAM20582	*Papilio polytes*	Specialist	Solanaceae	Serine protease	KDSCQGDS	Epidermis
126	KPJ14530	*Papilio machaon*	Specialist	Solanaceae	Transmembrane protease serine 9	KDACTGDS	Whole organism
127	OWR48668	*Danaus plexippus plexippus*	Specialist	Apocynaceae	serine protease protein precursor	KDACTGDS	Whole organism
128	XP_022113517	*Pieris rapae*	Specialist	Crucifers	Trypsin-1-like isoform X1	TDSCQGDS	Whole organism
129	XP_022113520	*Pieris rapae*	Specialist	Crucifers	Trypsin-1-like	KDACQGDS	Whole organism
130	XP_022113521	*Pieris rapae*	Specialist	Crucifers	Serine protease 42-like	KDACTGDS	Whole organism
131	XP_022113599	*Pieris rapae*	Specialist	Crucifers	Transmembrane protease serine 9-like	KDACTGDS	Whole organism
132	XP_022114790	*Pieris rapae*	Specialist	Crucifers	Venom protease-like	KDACHADS	Whole organism
133	XP_022115694	*Pieris rapae*	Specialist	Crucifers	Atrial natriuretic peptide-converting enzyme-like	KDTCQGDS	Whole organism
134	XP_022116699	*Pieris rapae*	Specialist	Crucifers	Uncharacterized threonine-rich GPI-anchored glyco PJ4664.02-like	RDSCAGDS	Whole organism
135	XP_022117181	*Pieris rapae*	Specialist	Crucifers	Vitamin K-dependent C	MDSCSGDS	Whole organism
136	XP_022117558	*Pieris rapae*	Specialist	Crucifers	Proclotting enzyme	KDSCWADS	Whole organism
137	XP_022118165	*Pieris rapae*	Specialist	Crucifers	Coagulation factor XII-like isoform X1	RDSCQGDS	Whole organism
138	XP_022118171	*Pieris rapae*	Specialist	Crucifers	Mucin-5AC	RDACQGDS	Whole organism
139	XP_022123051	*Pieris rapae*	Specialist	Crucifers	Proclotting enzyme-like isoform X2	EGTCQGDS	Whole organism
140	XP_022123529	*Pieris rapae*	Specialist	Crucifers	Venom serine protease-like	KDACQFDS	Whole organism

SN	Clone ID/Accesion#	Organism	Herbivore type	Host plant/Family	Descriptions in NCBI Annotations	8 amino-acid signature motif proximal to D189	Tissue source
141	XP_022123541	*Pieris rapae*	Specialist	Crucifers	Venom serine protease-like isoform X2	EGGCQGDS	Whole organism
142	XP_022124842	*Pieris rapae*	Specialist	Crucifers	Transmembrane protease serine 9-like	KDSCSGDS	Whole organism
143	XP_022126893	*Pieris rapae*	Specialist	Crucifers	Proclotting enzyme	KDSCQGDS	Whole organism
144	XP_022126894	*Pieris rapae*	Specialist	Crucifers	Serine protease 40 partial	QDACLGDS	Whole organism
145	XP_022127727	*Pieris rapae*	Specialist	Crucifers	Serine proteinase stubble isoform X1	HDSCQGDS	Whole organism
146	XP_022130881	*Pieris rapae*	Specialist	Crucifers	Serine proteinase stubble	SDSCEGDS	Whole organism
147	XP_023954105	*Bicyclus anynana*	Specialist	Poaceae	Trypsin-7-like	KDACTGDS	Larval whole body
**148**	**ASF/AHA11074**	***Pieris brassicae***	**Specialist**	**Crucifers**	**Putative serine proteinase, partial**	**KDACQGDS**	**Gut**
**149**	**DSH22/QDX19125**	***Pieris brassicae***	**Specialist**	**Crucifers**	**Putative serine protease, partial**	**KGACYGDS**	**Gut**
150	XP_022112767	*Pieris rapae*	Specialist	Crucifers	Chymotrypsin-1-like	HGVCNGDS	Whole organism
151	XP_022112774	*Pieris rapae*	Specialist	Crucifers	Chymotrypsin-1-like	HGMCHGDS	Whole organism
152	XP_022112832	*Pieris rapae*	Specialist	Crucifers	Chymotrypsin-1-like	YGTCNGDS	Whole organism
153	XP_022112833	*Pieris rapae*	Specialist	Crucifers	Chymotrypsin-1-like	HGTCNGDS	Whole organism
154	XP_022112835	*Pieris rapae*	Specialist	Crucifers	Chymotrypsin-1-like	QGTCNGDS	Whole organism
155	XP_022115401	*Pieris rapae*	Specialist	Crucifers	Chymotrypsin-2-like	VDTCQGDS	Whole organism
156	XP_022115886	*Pieris rapae*	Specialist	Crucifers	Chymotrypsin-1-like	KDACQGDS	Whole organism
157	XP_022118013	*Pieris rapae*	Specialist	Crucifers	Trypsin-5-like	RDSCQGDS	Whole organism
158	XP_022119534	*Pieris rapae*	Specialist	Crucifers	Snake venom serine protease bmsp-like	RDQCGGDS	Whole organism
159	XP_022123055	*Pieris rapae*	Specialist	Crucifers	Chymotrypsin-1	KDTCQGDS	Whole organism
160	XP_022123383	*Pieris rapae*	Specialist	Crucifers	Chymotrypsin-2-like	KDACQFDS	Whole organism
161	XP_022123773	*Pieris rapae*	Specialist	Crucifers	Chymotrypsin-1-like	EGICQGDS	Whole organism
162	XP_022123774	*Pieris rapae*	Specialist	Crucifers	Chymotrypsin-2-like	KDSCQGDS	Whole organism
163	XP_022124211	*Pieris rapae*	Specialist	Crucifers	Trypsin-2-like	KDACNHDS	Whole organism
164	XP_022124347	*Pieris rapae*	Specialist	Crucifers	Chymotrypsin-2-like isoform X1	KGACYGDS	Whole organism
165	XP_022124469	*Pieris rapae*	Specialist	Crucifers	Chymotrypsin-2-like	KGACYGDS	Whole organism
166	XP_022124470	*Pieris rapae*	Specialist	Crucifers	Chymotrypsin-2-like	KGACQGDS	Whole organism
167	XP_022124474	*Pieris rapae*	Specialist	Crucifers	Transmembrane protease serine 9-like	KGACKGDS	Whole organism
168	XP_022124475	*Pieris rapae*	Specialist	Crucifers	Chymotrypsin-2-like	EGACQGDS	Whole organism
169	XP_022124477	*Pieris rapae*	Specialist	Crucifers	Chymotrypsin-2-like	KGTCKGDS	Whole organism

SN	Clone ID/Accesion#	Organism	Herbivore type	Host plant/Family	Descriptions in NCBI Annotations	8 amino-acid signature motif proximal to D189	Tissue source
170	XP_022124554	*Pieris rapae*	Specialist	Crucifers	Chymotrypsin-2-like	NNTFQGDS	Whole organism
171	XP_022124751	*Pieris rapae*	Specialist	Crucifers	Chymotrypsin	EGACHGDS	Whole organism
172	XP_022124753	*Pieris rapae*	Specialist	Crucifers	Chymotrypsin-2-like	KGICRGDS	Whole organism
173	XP_022124754	*Pieris rapae*	Specialist	Crucifers	Chymotrypsin-2-like	RDSCTGDS	Whole organism
174	XP_022126022	*Pieris rapae*	Specialist	Crucifers	Chymotrypsin-1-like	EGTCQGDS	Whole organism
175	XP_022126482	*Pieris rapae*	Specialist	Crucifers	Chymotrypsin-2-like	EGTCQGDS	Whole organism
176	XP_022126878	*Pieris rapae*	Specialist	Crucifers	Chymotrypsin-2-like	QGTCQGDS	Whole organism
177	XP_022126886	*Pieris rapae*	Specialist	Crucifers	Chymotrypsin-1-like	EGTCQGDS	Whole organism
178	XP_022127224	*Pieris rapae*	Specialist	Crucifers	Chymotrypsin-2-like	QGFCQGDS	Whole organism
179	XP_022127751	*Pieris rapae*	Specialist	Crucifers	Chymotrypsin-2-like	GSWMEGDS	Whole organism
180	XP_022127752	*Pieris rapae*	Specialist	Crucifers	Chymotrypsin-1-like	TGGCKGDS	Whole organism
181	XP_022127836	*Pieris rapae*	Specialist	Crucifers	Chymotrypsin-2-like	EGSCQGDS	Whole organism
182	XP_022127838	*Pieris rapae*	Specialist	Crucifers	Chymotrypsin-2-like	KGACHGDS	Whole organism
183	XP_022128680	*Pieris rapae*	Specialist	Crucifers	Chymotrypsin-2-like	KGACHGDS	Whole organism
184	XP_022128681	*Pieris rapae*	Specialist	Crucifers	Chymotrypsin-2-like	SGACKGDS	Whole organism
185	XP_022128682	*Pieris rapae*	Specialist	Crucifers	Chymotrypsin-2-like	TGACHGDS	Whole organism
186	XP_022128683	*Pieris rapae*	Specialist	Crucifers	Chymotrypsin-1-like	TGGCKGDS	Whole organism
187	XP_022128928	*Pieris rapae*	Specialist	Crucifers	Chymotrypsin-1-like	EGFCQGDS	Whole organism
188	XP_022129001	*Pieris rapae*	Specialist	Crucifers	Chymotrypsin-1-like	EGTCQGDS	Whole organism
189	XP_022129217	*Pieris rapae*	Specialist	Crucifers	Chymotrypsin-2-like	SGACKGDS	Whole organism
190	XP_022129219	*Pieris rapae*	Specialist	Crucifers	Chymotrypsin-2-like	KGTCRGDS	Whole organism
191	XP_022129220	*Pieris rapae*	Specialist	Crucifers	Low quality chymotrypsin-1-like	EGACHGDS	Whole organism
192	XP_022129222	*Pieris rapae*	Specialist	Crucifers	Chymotrypsin-2-like	QGTCQGDS	Whole organism
193	XP_022129223	*Pieris rapae*	Specialist	Crucifers	Chymotrypsin-2-like	KGACHGDS	Whole organism
194	XP_022113091	*Pieris rapae*	Specialist	Crucifers	Chymotrypsin-1-like	RAVCSGDS	Whole organism
195	XP_022115254	*Pieris rapae*	Specialist	Crucifers	Serine protease gd-like	EGTCQGDS	Whole organism
196	XP_022115755	*Pieris rapae*	Specialist	Crucifers	Trypsin-1-like	EGACHGDS	Whole organism
197	XP_022118008	*Pieris rapae*	Specialist	Crucifers	Transmembrane protease serine 9-like	PGMCNGDS	Whole organism
198	XP_022120055	*Pieris rapae*	Specialist	Crucifers	Serine protease snake-like	KDSCKGDS	Whole organism

SN	Clone ID/Accesion#	Organism	Herbivore type	Host plant/Family	Descriptions in NCBI Annotations	8 amino-acid signature motif proximal to D189	Tissue source
199	XP_022120772	*Pieris rapae*	Specialist	Crucifers	Serine protease gd-like	KDACQGDS	Whole organism
200	XP_022125018	*Pieris rapae*	Specialist	Crucifers	Chymotrypsin	TTLCKGDS	Whole organism
201	XP_022126007	*Pieris rapae*	Specialist	Crucifers	Modular serine protease-like isoform X2	EGTCQGDS	Whole organism
202	XP_022127469	*Pieris rapae*	Specialist	Crucifers	Serine protease snake-like	KDSCEGDS	Whole organism
203	XP_022127723	*Pieris rapae*	Specialist	Crucifers	Chymotrypsin-1-like	RDTCKGDS	Whole organism
204	XP_022128447	*Pieris rapae*	Specialist	Crucifers	Venom protease-like	KDTCQGDS	Whole organism
205	XP_022128678	*Pieris rapae*	Specialist	Crucifers	Chymotrypsin-2-like	SGACKGDS	Whole organism
206	XP_022130158	*Pieris rapae*	Specialist	Crucifers	Modular serine protease-like	TTLCKGDS	Whole organism
207	XP_022130765	*Pieris rapae*	Specialist	Crucifers	Serine protease 41-like	TSPLQGDS	Whole organism
208	XP_022130766	*Pieris rapae*	Specialist	Crucifers	Serine protease 41-like	GTISRGDS	Whole organism
