# Supplementary material for: Consequences of ‘no-choice, fixed time’ reciprocal host plant switches on nutrition and gut serine protease gene expression in Pieris brassicae L. (Lepidoptera: Pieridae)
Source: PLoS One. 2021 Jan 20;16(1):e0245649. doi: 10.1371/journal.pone.0245649 (PMC7817030; doi:10.1371/journal.pone.0245649)
Supplement: S1 File — (PDF) [file pone.0245649.s006.pdf]

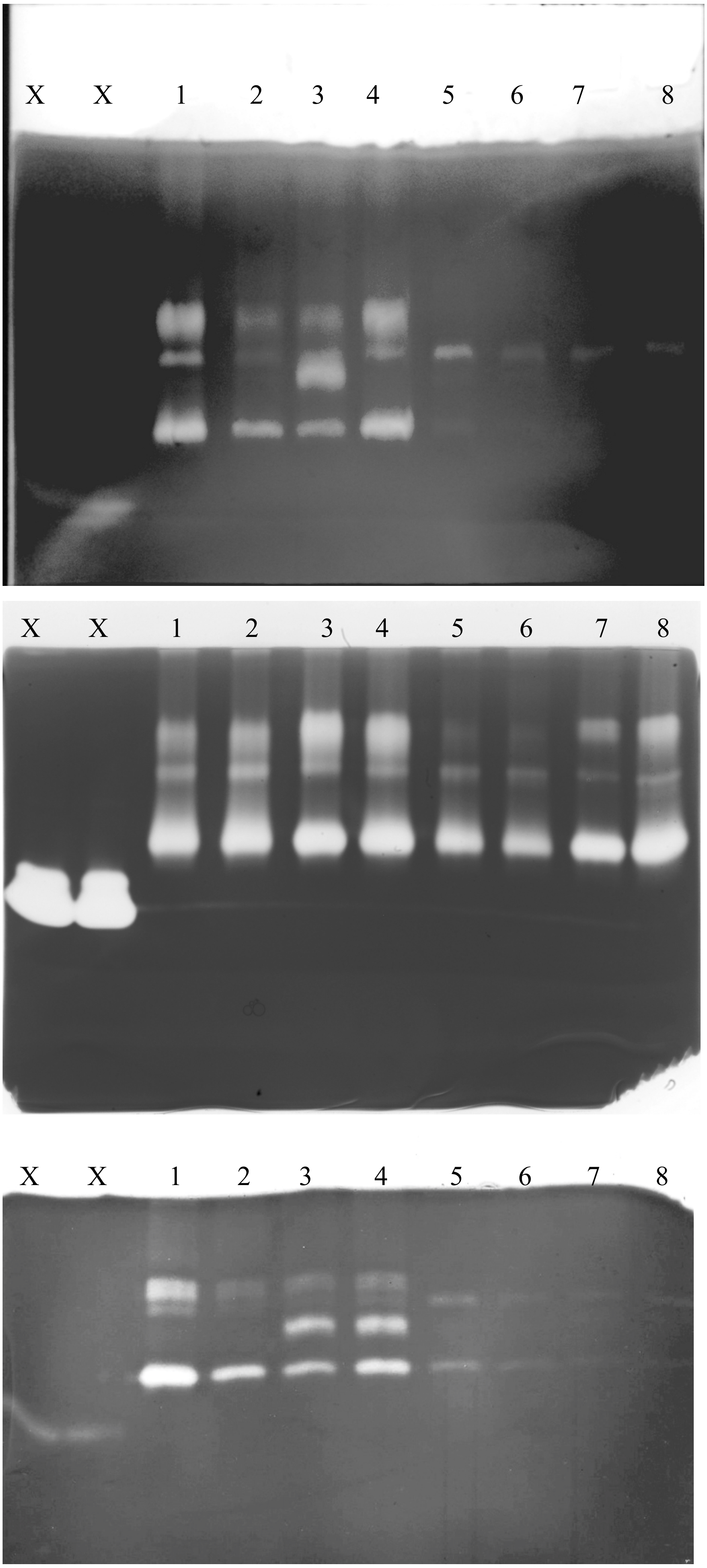

Fig 4: Raw gel pictures showing 12% gelatinolytic zymograms showing protease activities in gut extracts of *P. brassicae* larvae of different ages fed on different diets. Panels show results for (A) III instars, (B) IV instars, and (C) V instars, feeding on (lane 1) CF-CF, (lane 2) CF-TP, (lane 3) TP-TP and (lane 4) TP-CF diets, while lanes 5-8 correspond to the same samples incubated with 5mM TLCK. Lanes X refers to lanes not included in the final figure.

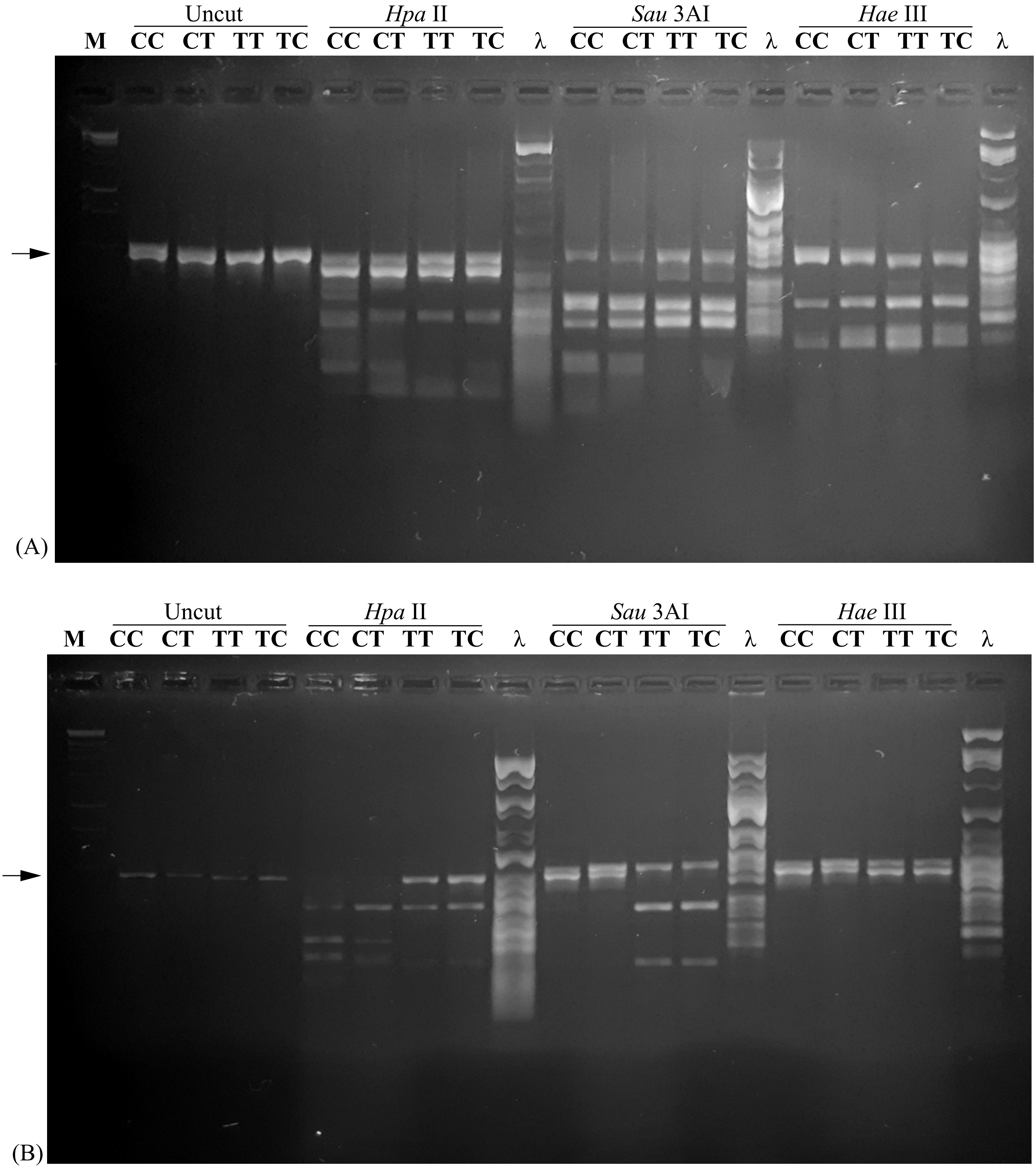

S2 Fig A-B: A 2.5% agarose gel showing *Hpa* II, *Sau* 3AI and *Hae* III digested RT-PCR products amplified from gut tissues of larvae fed on CF-CF (CC), CF-TP (CT), TP-TP (TT) and TP-CF (TC) diets using serine protease-specific primer pairs: (A) DmTF/R and (B) DmTF/SerPR. Lanes M show 1 kb ladder (Fermentas, USA, catalog# SM0312), and lanes λ show lambda DNA digestion products. Arrows indicate product of ~500bp.

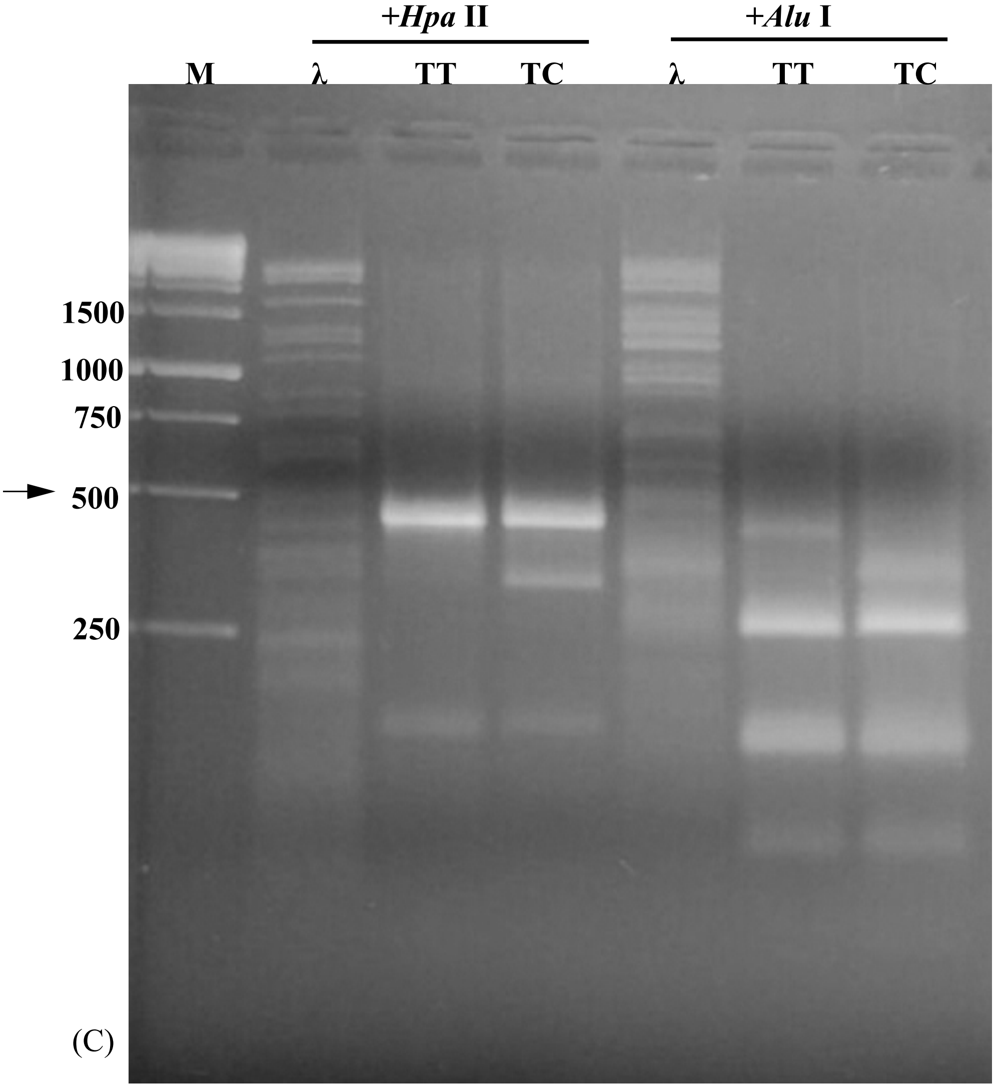

S2 Fig C: A 2.5% agarose gel showing *Hpa* II, and *Alu* I digested products amplified with DmTF/R from larvae fed on TT and TC diets. Lanes M show 1 kb ladder (Fermentas, USA, catalog# SM0312), and lanes λ show lambda DNA digestion products. Arrows indicate product of ~500bp.
